# Effects of damage initiation points of depth-damage function on flood risk assessment

**DOI:** 10.1038/s44304-024-00004-z

**Published:** 2024-05-02

**Authors:** Md Adilur Rahim, Ayat Al Assi, Rubayet Bin Mostafiz, Carol J. Friedland

**Affiliations:** 1 LaHouse Resource Center, Department of Biological and Agricultural Engineering, Louisiana State University Agricultural Center, Baton Rouge, LA USA; 2 Engineering Science Program, Louisiana State University, Baton Rouge, LA USA; 3 Coastal Studies Institute, Louisiana State University, Baton Rouge, LA USA; 4 Bert S. Turner Department of Construction Management, Louisiana State University, Baton Rouge, LA USA

**Keywords:** Natural hazards, Water resources

## Abstract

The flood depth in a structure is a key factor in flood loss models, influencing the estimation of building and contents losses, as well as overall flood risk. Recent studies have emphasized the importance of determining the damage initiation point (DIP) of depth-damage functions, where the flood damage is assumed to initiate with respect to the first-floor height of the building. Here we investigate the effects of DIP selection on the flood risk assessment of buildings located in Special Flood Hazard Areas. We characterize flood using the Gumbel extreme value distribution’s location (*μ*) and scale (*α*) parameters. Results reveal that average annual flood loss (AAL) values do not depend on *μ*, but instead follow an exponential decay pattern with *α* when damage initiates below the first-floor height of a building (i.e., negative DIP). A linear increasing pattern of the AAL with *α* is achieved by changing the DIP to the first-floor height (i.e., DIP = 0). The study also demonstrates that negative DIPs have larger associated AAL, thus contributing substantially to the overall AAL, compared to positive DIPs. The study underscores the significance of proper DIP selection in flood risk assessment.

## Introduction

Flood depth-damage functions (DDFs) are valuable in providing rapid estimates of economic loss as a function of flood depths^1–5^. DDFs are developed by one of two primary methods—through analysis of actual flood insurance claims data (empirical); or through analysis of probable loss as a function of depth as determined by computational analysis or a group of experts (synthetic)^6,7^. Synthetic DDFs are also often developed through expert analysis^8–10^ and then validated by actual flood loss data^11,12^. While these functions are known in the literature as “depth-damage” functions, they actually express the loss sustained by a building, generally represented as the economic loss as a percentage of building replacement value (BRV)^1^. DDFs provide valuable loss estimates for individual buildings and communities in actual flood events or for planning purposes^13^, and often serve as the backbone of benefit quantification in flood mitigation benefit-cost analyses^14,15^.

Several sources of DDFs are available, but the most widely used in the U.S.A. are those developed by the U.S. Army Corps of Engineers (USACE) and by the National Flood Insurance Program (NFIP), formerly known as the Flood Insurance Agency (FIA), managed by the Federal Emergency Management Agency (FEMA) Mitigation Directorate^16^. It’s worth noting that these DDFs are applied consistently on a national level and have even been integrated into FEMA’s HAZUS-MH software. Moreover, their impact extends globally, as they serve as fundamental tools for flood risk assessment and mitigation strategies in various regions^17^.

As mentioned in FEMA^18^, FEMA DDFs were developed based on claims submitted to the National Flood Insurance Program (NFIP). FIMA (formerly known as FIA) oversees the NFIP and initially created depth-damage curves in the early 1970s, incorporating insights from surveys conducted by the Corps of Engineers following floods. These original curves, known as “theoretical base tables,” provided a foundation. Over time, an extensive data set of flood-related losses, encompassing both structural and content damage and calculated against the actual cash value, has been accumulated through flood insurance claims. These claims predominantly involve residential structures. The FEMA DDFs were primarily based on data from the period of 1978 to 1997, as indicated by the “Depth-Damage” report prepared by the NFIP Actuarial Information System in 1998. It’s important to note that the final damage functions incorporated into the current version of the Hazus Flood Model software, which is an extension of FEMA’s work, are based on FIA data through 2001.

Under NFIP coverage, “substantial damage” is considered to occur when loss equals or exceeds approximately 50% of the total BRV. Structures that sustain a loss that exceeds 50% typically are considered a “total loss.” As a result, many of the FEMA DDFs reflect coverage limitations in the NFIP and not necessarily the actual costs that result from the flood event. FEMA^19^ states that the USACE DDFs may better represent actual losses associated with flooding events because the underlying data for these functions were collected with the intent of representing the total loss without regard to NFIP coverage.

The USACE^20^ DDFs are based on loss data collected in major flood events in the U.S.A. from 1996 to 1998^14,20^. The USACE^21^ DDFs were revised to account for the significant floods that occurred in the U.S.A. between 1996 and 2001^21^. These DDFs exhibit several key characteristics that highlight their adaptability. Firstly, DDFs exhibit spatial variability, recognizing that flood risk and building characteristics differ across geographical regions. For instance, the USACE^22^ DDFs for Louisiana’s coast showcases how DDFs were developed to address the specific challenges of Southern Louisiana, considering factors unique to that region, such as building types and vulnerabilities associated with recurrent flooding. This development involved input from homeowners, business operators, and experts in construction, repair, restoration, and insurance claims adjustment. Similarly, the USACE^23^ coastal DDFs for the North Atlantic Coast demonstrate the importance of customizing DDFs for regions with distinct characteristics, including high-rise structures and coastal storm impacts. These DDF were developed based on post-Hurricane Sandy damage surveys and expert elicitation. Secondly, DDFs distinguish between different types of buildings, recognizing that the extent of damage varies based on structural features. USACE^23^, for example, identifies ten typical structure groups within the North Atlantic region, illustrating how DDFs categorize buildings to provide more accurate damage assessments. Lastly, DDFs consider various flood characteristics, such as rate of rise, water depth, wave height, and duration, as seen in USACE^23^. Similarly, the USACE^22^ DDFs for Louisiana’s coast were formulated for four hydrologic conditions, including short-duration and long-duration freshwater flooding due to riverine or rainfall events, and short-duration and long-duration saltwater flooding due to hurricanes. These factors enable DDFs to accommodate diverse flood scenarios, ensuring precision accuracy in damage estimation.

The damage initiation point (DIP)—the water depth at which a building begins incurring damage) of DDFs is an important parameter of choice that affects the estimation of flood loss and subsequently, flood risk of a structure. The DIP is calculated with respect to the first-floor height (FFH) of a building. For example, a DIP value of –2 feet means the damage to the building will start incurring when the flood water is –2 feet below the FFH. It’s important to note that DIP values vary depending on the specific DDF chosen for estimating the loss of the structure. Different DDFs have different DIP values, and these values can be influenced by factors such as building type, and geographic location. For example, consider the DDFs utilized by key institutions, the USACE^21^ DDF has a DIP value of –2 feet, while the FIA^24^ DDF has a DIP value of –1 foot, and the FEMA^18^ DDF has a DIP value of 0 feet. Additionally, certain DDFs, like the one developed by the Wing et al.^17^ adopt a DIP of 0 ft relative to FFH. Conversely, the DDF presented by Nofal et al.^25^ employs a more conservative DIP of −3 ft relative to FFH.

Furthermore, it’s worth noting that several factors affect the selection of DIPs, such as the type of foundation and the relative location and elevation of machinery and equipment (M&E) within a building. Foundation type can offer valuable information on the probable starting point of flood risk. Buildings that are elevated using enclosures rather than supported by from posts, piles, or piers will have a greater flood risk than elevated buildings that lack enclosure but are supported by posts, piles, or piers^26^. The DIP would change based on the foundation type (e.g., slab-on-grade, crawlspace) of a building^22,25^ and the relative location (i.e., inside or outside of home) and elevation with FFH of M&E^26^. For slab-on-grade foundations, the flood damage would start when the flood water is at (i.e., DIP = 0)^25^ the building’s FFH or near (i.e., DIP = –0.5)^22^ FFH if M&E are located above the FFH. The DIP of zero or –0.5 represents such a case in this study, and the results of this study assuming that DIP equals zero and –0.5 would be appropriate for these buildings. A negative DIP (i.e., –2, –1) is appropriate based on the foundation type (e.g., crawlspace)^25^ for M&E placed below the FFH. The positive and negative DIPs explored in this study facilitate the variability in AAL compared to the AAL estimated using the original DDF.

The variation in DIP of DDFs will directly affect the flood loss estimates and, subsequently, the flood risk assessment. For example, Gnan et al.^27^ demonstrated that flood risk in terms of average annual loss (AAL) derived from USACE^21,22^, Nofal et al.^25^, and Wing et al.^17^ functions that initiate damage at a DIP of –2 feet were much higher than AAL calculated from the same functions with damage initiation assumed to occur at 0 feet. Al Assi et al.^28^ demonstrated that AAL is increased fivefold to sevenfold if the DIP is considered at –1 foot compared to 0 feet.

This study examines the effects of DIPs on flood risk assessment of single-family one-story no-basement buildings located in special flood hazard area (SFHA—areas expected to experience a 1% or greater annual chance of flooding). The analysis utilizes the USACE^21^ DDF, assuming a datum of the top of the finished floor as the logical choice for one-story homes without basements. It is worth noting that the flood depth datum differs for FEMA functions, with A-zone measurements relative to the top of the lowest floor, while V-zone measurements are relative to the bottom of the lowest structural member. In this study, the selection of USACE^21^ DDF is motivated by their utilization of generic data from 1996 to 2001, thus making them suitable for showcasing the methodology^29^. The contributions of this research are the novel visual and quantitative representation of the impact of DIP selection on flood risk assessment, providing a comprehensive understanding of how DIP influences AAL for buildings located in SFHA. The relationship described between AAL and the flood hazard scale parameter for different DIPs can provide valuable insights into the relationship between these variables. Also, the effectiveness of freeboards in reducing flood risk for different DIP ranges was quantified, providing valuable assessments of the effect of freeboard as a flood risk reduction strategy.

## Results

### Flood hazard characterization

**Fig. 1:**
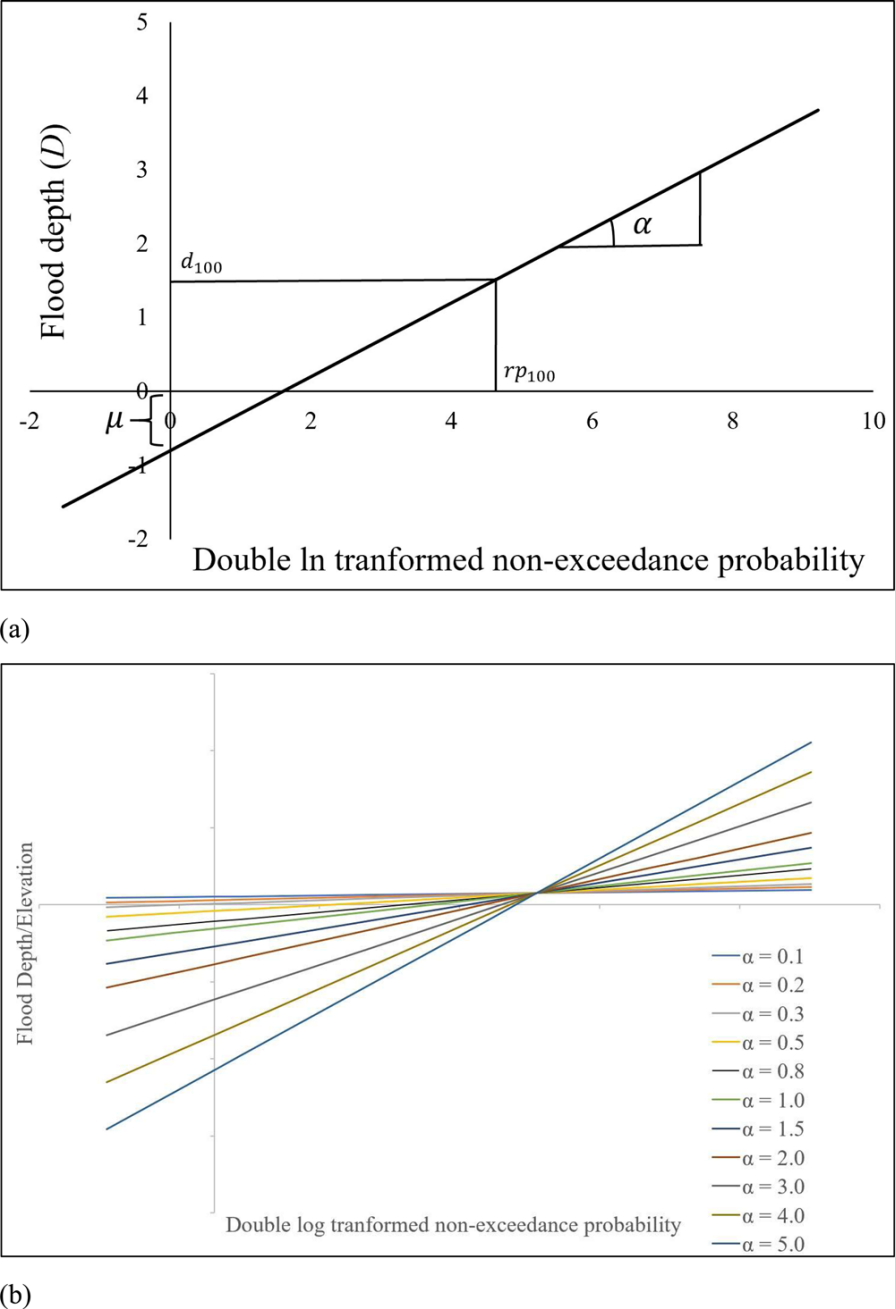
Flood characteristics with Gumbel extreme value distribution. **a** Straight line showing the location (*μ*) and scale (*α*) parameter. **b** Gumbel lines of buildings with FFH at ${d}_{100}$.

The flood hazard of a building is characterized by Gumbel location (*μ*) and scale (*α*) parameters (see Methods). The Gumbel distribution is fitted using flood depth/elevation data of a building. The *μ* and *α* parameters are the intercept and the slope value of the regression line fitted using a least-squares regression where the dependent variable is the flood hazard intensity and the independent variable is the double log-transformed non-exceedance probability of the flood events (Fig. 1a). In Fig. 1a, the ${d}_{100}$ is the base flood depth (BFD) of the building which represents the flood depth corresponding to ${{rp}}_{100}$ (i.e., 100-year return period flood) with exceedance probability of 0.01.

Figure 1b visualizes Gumbel lines of buildings with the FFH at ${d}_{100}$. The FFH of a building is measured as the height from adjacent grade of top of lowest floor for A zones, and bottom of the lowest horizontal structural member for V zones. In the United States, for buildings in SFHA, the FFH should be situated at least to BFE (i.e., 1-percent exceedance probability flood event). So, the FFH for these buildings are fixed to ${d}_{100}$ for no freeboard scenario. It is worth mention that FEMA designates the 100-year floodplain, also known as the SFHA, as regions with one percent or greater probability of annual flooding, whether inland or along the coast. These areas are identified on Flood Insurance Rate Maps (FIRMs) with labels beginning with either “A” or “V,” signifying their status as high-risk flood zones. The *α* value of the building represents the scale of the flood, where buildings with higher *α* values are exposed to greater intensity of higher return period flood events than buildings with lower *α*. Additionally, the *μ* parameter of the building represents the location of the flood, where a positive *μ* indicates that the building is located in an area prone to intense floods, while a negative *μ* value indicates the opposite. The *μ* parameter represents the $(\frac{e}{e-1})$ -year return period flood elevation at a location, which is directly related to ground elevation. For example, *μ* would be positive for locations in coastal areas or water bodies, and negative for locations in non-water bodies, such as residential areas (Mostafiz et al.^30^). The combination of both *μ* and *α* parameters provides a more comprehensive understanding of the characteristics of flood events and their severity.

### Flood hazard and risk relationship

The AAL value for buildings with known hazard parameters is estimated using a Monte Carlo simulation. The simulation is run for different *α*, BFD, and DIP values to estimate the associated AAL as a percentage of the building replacement value. The *μ* parameter is calculated using the *α* and BFD values from the quantile of the Gumbel distribution. The generated data set covers buildings located in the SFHA (see Methods).

**Fig. 2:**
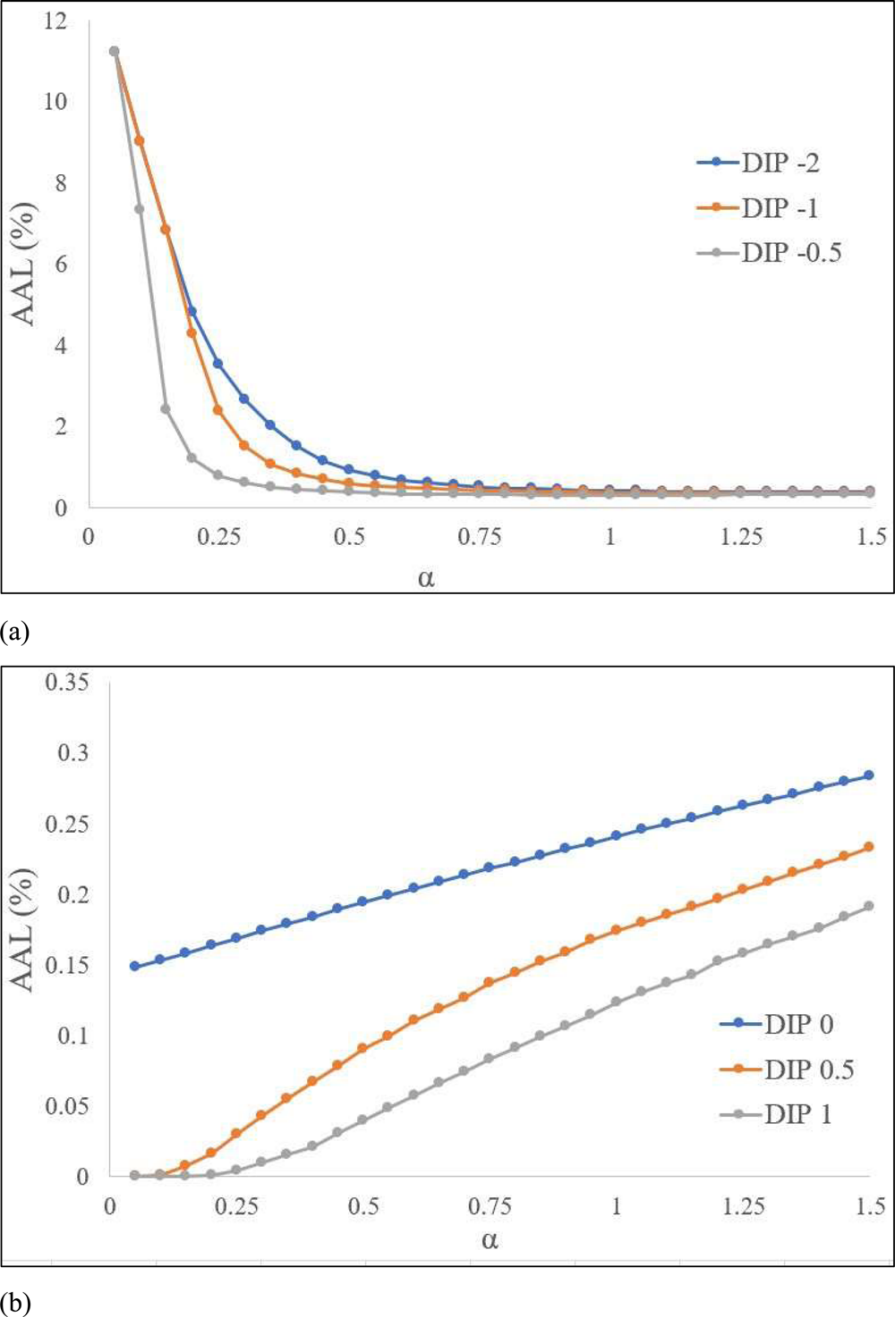
Pattern of flood risk with hazard scale parameter using USACE (2003) structure DDF. **a** AAL (%) curves for DIPs below first floor. **b** AAL curves for DIPs at or above first floor.

The data analysis shows that varying BFD values have no effect on the calculation of AAL percentages. In practical terms, whether the BFD is set at 2 feet or 12 feet, the AAL (%) for different *μ* values do not change (Supplementary Fig. S.1). Consequently, in scenarios where the *α* parameter remains constant, the AAL (%) is unaffected by variations in the *μ* parameter for a specific DIP. However, changes in the *α* parameter do impact the AAL (%), and this relationship depends on the DIP value (Supplementary Tables S.1–S.5). For negative DIP values, the AAL (%) value decreases with increasing *α*, and for zero to positive DIP values, the AAL (%) value linearly increases with increasing *α*. Looking into the relationship between risk and hazard in the “no freeboard” scenario, the negative DIPs have an exponential decay trend of AAL (%) with increasing *α* (Fig. 2a), where the positive DIPs have a linear increasing trend (Fig. 2b).

In this study, general equations for the DIP values of –2 and 0 are explored and developed. In the case of DIP –2, the residual plots show the trends to be nonlinear. A simple way to fit a nonlinear trend is to use quadratic or higher-order trends. However, this is not recommended as they are not good at forecasting extrapolated values. Here, a piecewise linear approach, where knots are introduced to mark the change in the slope of the curve and the nonlinear trend can be constructed using linear trends, is undertaken. As the AAL (%) curve exponentially decays up to *α* value of 0.7, a knot at 0.7 is introduced, and two models are fit to represent this curve. An exponential decay trend which is equivalent to a log-linear regression in which the AAL (%) values are log-transformed for *α* values ≤0.7 is fit. The model provides a good fit with adjusted *R*^2^ value of 0.9773. A quadratic model is fit for *α* values >0.7 with adjusted *R*^2^ value of 0.9813. The equation of the fitted models is given below:

$${AAL}\left( \% \right)=12.691{e}^{-4.9135\alpha }{{;}}\,\alpha \le 0.7$$

$${AAL}\left( \% \right)=0.3975{\alpha }^{2}-1.0602\alpha +1.0846{{;}}\,0.7 \,<\, \alpha \le 1.5$$

In the case of DIP 0, the AAL (%) curve shows an increasing trend, and the residual plot shows the trend to be nonlinear. An exponential function which provides an adjusted *R*^2^ value of 0.9847 is then fit. The equation is provided below:

$${AAL}\left( \% \right)=0.153{e}^{0.4389\alpha }$$

The AAL (%) curves shows an increasing trend with increasing *α* parameter for different DIPs as a result of adding freeboard with the exception of the freeboard +1 feet and DIP –2 scenario (Supplementary Fig. S.2). Implementing +1 feet of freeboard reduces the maximum AAL (%) from 11.21% (the “no freeboard” scenario) to 2.002% for a DIP of –2. General equations of AAL (%) and *α* parameter for a +1 feet of freeboards and DIPs of –2 (nonlinear) and 0 (linear) feet where *α* ranges from 0.05 to 1.5 are given below.

$${AAL}\left( \% \right)=2.949{e}^{-7.918\alpha }{{;}}\,\alpha \le 0.4\,[{R}^{2}:0.9743]$$

$${AAL}\left( \% \right)=0.762{\alpha }^{2}-0.0954\alpha +0.1712{{;}}\,0.4 \,<\, \alpha \le 1.5\,[{R}^{2}:0.9429]$$

$${AAL}\left( \% \right)=0.1193\alpha -0.0324\,[{R}^{2}:0.9994]$$

**Fig. 3:**
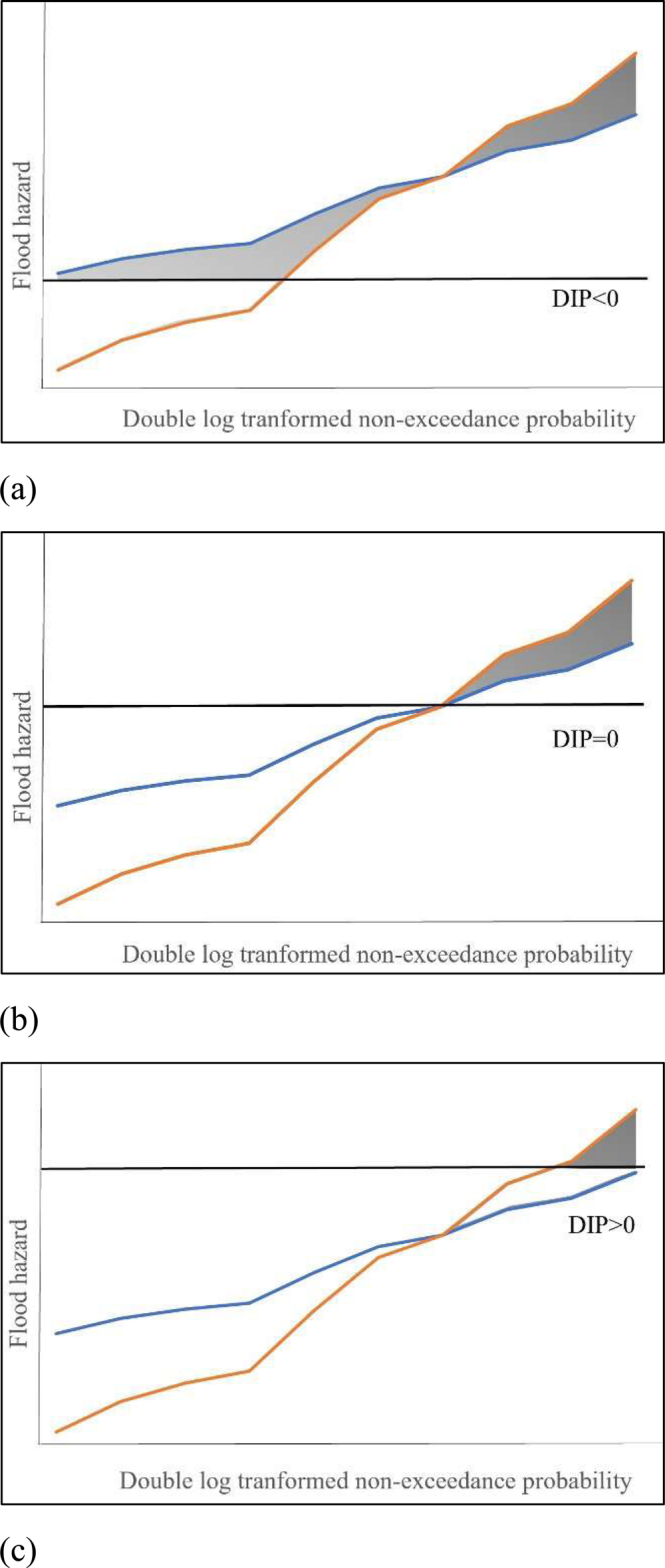
The effect of DIP in flood hazard selection visualized using Gumbel lines for two buildings where higher *α* line is represented in orange and lower *α* line is represented in blue. **a** DIP is below FFH; shaded region contains both low and high probability flood events. **b** DIP is at FFH; neglects high probability flood events and only considers low probability flood events in estimating AAL (%). **c** DIP is above FFH; partly considers low probability flood events in estimating AAL (%).

The effect of DIP in the flood risk pattern for buildings in the SFHA with different hazard characteristics but FFHs are at ${d}_{100}$ or at same exceedance probability can be described using Fig. 3. It shows the Gumbel lines for two buildings with FFH (i.e., the point where the lines intersect) at same exceedance probability. Figure 3a presents the scenario when the DIP is below the FFH, while Fig. 3b, c present the scenarios when the DIP is at and above the FFH, respectively. The shaded region below the FFH represents the flood events with higher probabilities that the building with lower *α* (blue line) will experience in its lifetime, whereas the building with higher *α* (orange line) will not experience these events. The shaded region above the FFH (Fig. 3b) represents the flood events with lower probabilities that the higher *α* building may experience in its lifetime, whereas the lower *α* building will not experience these extreme events. When estimating the AAL (%) values for negative DIPs, the shaded region that consists of the high probability flood events contributes to the total AAL (%) for lower *α* value buildings, and the shaded region that consists of the low probability flood events contributes to the total AAL (%) for higher *α* value buildings. When the DIP is at the first floor (DIP = 0) or above, there is no shaded region of high probability floods, and only the shaded region with lower probability flood events contributes to the total AAL (%) for higher *α* value buildings. So, when the DIP is negative, the AAL (%) value is greater for lower *α* and when the DIP is zero or positive, the AAL (%) value is greater for higher *α*.

**Fig. 4:**
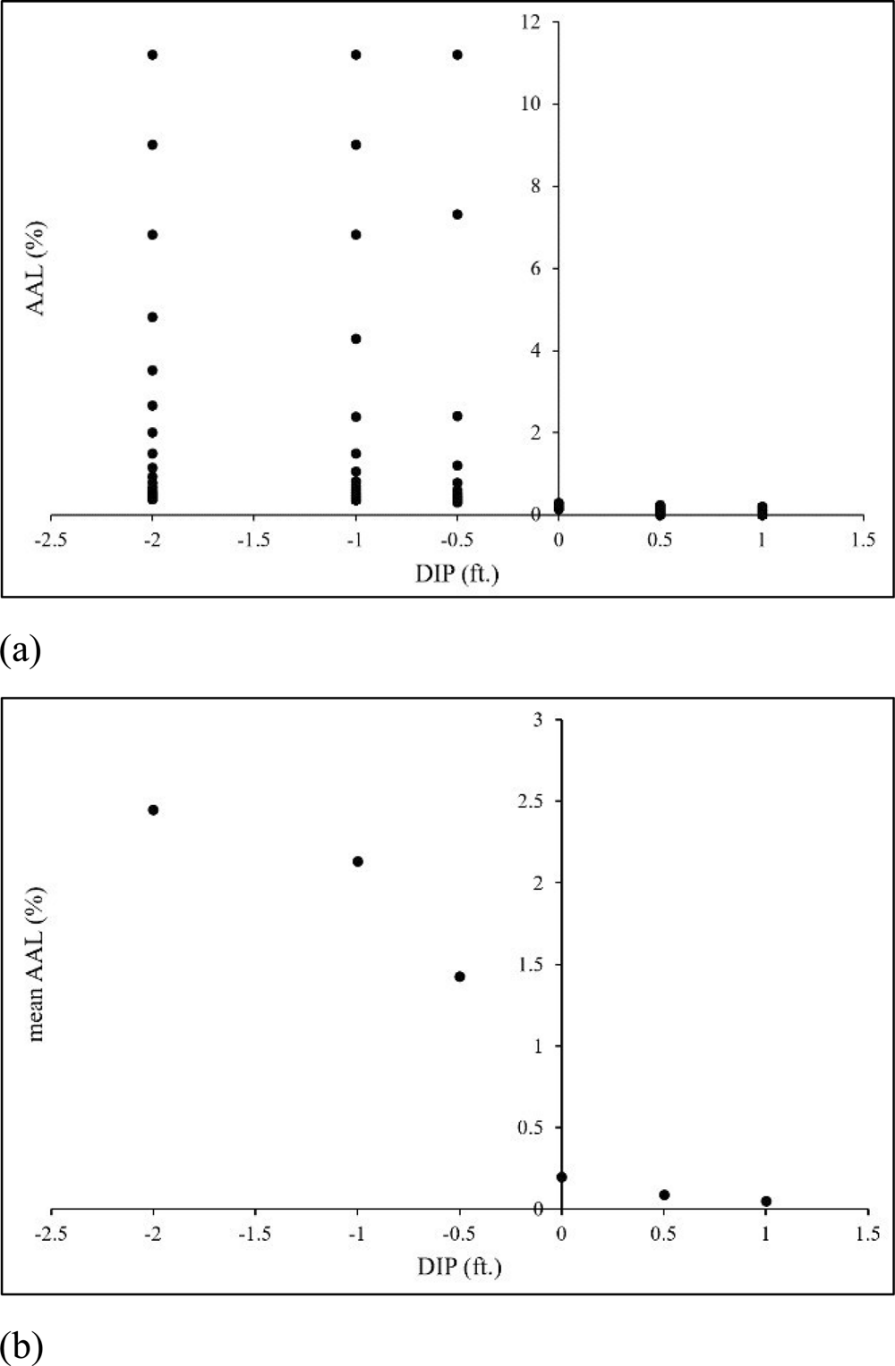
Result for no freeboard scenario. **a** Scatterplot of DIPs and associated AAL (%) values. **b** mean values of AAL (%) for each DIP.

### Sensitivity of DIP selection on AAL reduction by freeboard

The scatterplot of the data for the “no freeboard” scenario is presented in Fig. 4a. It shows the AAL (%) values for different DIPs. The estimated AAL (%) values are higher for negative DIPs than positive DIPs as expected. The spread of AAL (%) for negative DIPs is similar, as is the scatter for positive DIPs. However, there are differences in the mean AAL (%) values. Figure 4b demonstrates the reduction in mean AAL (%) with increasing DIP values. The relative and absolute reduction of mean AAL (%) with increasing DIP for the “no freeboard” scenario is presented in Table 1 and Table 2, respectively. There is a significant 86.3% reduction in mean AAL (%) when the DIP value moves from –0.5 feet to 0 feet, as shown in Table 1. A reduction of 92% in mean AAL (%) is calculated when DIP value moves from –2 feet to the FFH of a building. It’s important to highlight that the total mean reduction in AAL (%) is calculated in Table 2, representing the overall reduction in mean AAL (%) achieved when shifting DIP from –2 to 0 value within the DDF. Specifically, in the context of the ‘no freeboard’ scenario, if a DIP of –2 is selected instead of 0, this cumulative reduction of 2.25 in mean AAL (%) should be added to the building’s AAL estimation.

**Table 1:** Relative percentage reduction in mean AAL (%) for different ranges of DIPs and freeboards

Range of DIP	Relative percentage reduction in mean AAL (%)				
	FB = 0	FB = 1	FB = 2	FB = 3	FB = 4
–2 to –1	12.88	77.80	27.44	20.52	16.91
–1 to –0.5	33.09	36.57	24.00	21.06	21.07
–0.5 to 0	86.28	36.69	32.57	28.45	24.43
0 to 0.5	54.55	38.36	34.75	34.42	27.54
0.5 to 1	44.38	40.07	35.50	31.20	33.06
–2 to 0	92.00	91.08	62.82	55.11	50.45

**Table 2:** Absolute reduction in mean AAL (%) for different ranges of DIPs and freeboards

Range of DIP	Absolute reduction of mean AAL (%)				
	FB = 0	FB = 1	FB = 2	FB = 3	FB = 4
–2 to –1	0.31550	0.29871	0.00653	0.00120	0.00029
–1 to –0.5	0.70612	0.03117	0.00415	0.00097	0.00030
–0.5 to 0	1.23178	0.01984	0.00428	0.00104	0.00027
0 to 0.5	0.10684	0.01313	0.00308	0.00090	0.00023
0.5 to 1	0.03951	0.00846	0.00205	0.00054	0.00020
Total: –2 to 0	2.25339	0.34971	0.01495	0.00321	0.00085

As, freeboard has a significant effect in reducing the flood risk of buildings, the relative percentage and absolute reduction in mean AAL (%) for increasing freeboard scenarios are also presented in Table 1 and Table 2, respectively. For a foot of freeboard, the mean AAL (%) reduction is 77.8% for DIP range of –2 to –1 and 91.1% for DIP range of –2 to 0. Compared with the “no freeboard” scenario in which the range of DIP from –2 to 0 feet contributes to 2.25 of total mean AAL (%), freeboard of +1, +2, +3, and +4 feet largely reduces this percentage of AAL, yielding values of 0.35, 0.015, 0.003, and 0.00085, respectively for the same range of DIP. In the data, although the AAL (%) for positive DIPs are estimated, it is highly unlikely that a building will have a positive DIP value. The maximum DIP value that a building can incur is zero (i.e., DIP is at FFH).

To provide further clarity regarding the influence of DIP selection on AAL estimates in terms of monetary values, Table 3 presents the absolute change in mean AAL ($) values for a hypothetical building with a replacement value of $250,000. Additionally, the impact of DIP on AAL estimation with *α* parameter is detailed in Table 4. The study considered *α* range from 0.05 to 1.5, resulting in AAL (%) values spanning from 11.21 to 0.379 (Supplementary Table S.1) and AAL ($) values ranging from $28,023 to $948 for DIP –2.

**Table 3:** Absolute mean AAL ($) reduction for different ranges of DIPs and freeboards for a hypothetical $250,000 home

Range of DIP	Absolute mean AAL ($) reduction				
	FB = 0	FB = 1	FB = 2	FB = 3	FB = 4
–2 to –1	789	747	16	3	1
–1 to –0.5	1765	78	10	2	1
–0.5 to 0	3079	50	11	3	1
Total: –2 to 0	5633	875	37	8	3

**Table 4:** Absolute mean AAL ($) reduction for different ranges of DIPs and freeboards for a hypothetical $250,000 home, considering the minimum and maximum α in this study

*α*	AAL ($) (DIP –2)	AAL ($) (DIP –1)	AAL ($) (DIP –0.5)	AAL ($) (DIP 0)	AAL ($) (DIP 0.5)	AAL ($) (DIP 1)
0.05	28023	28023	28023	370	0	0
1.5	948	893	820	709	582	478

## Discussion

DDFs are of the utmost importance in flood studies as they quantify the relationship between flood loss and flood depth. While using the common DDFs, the damage a building incurs through a flood event starts even when the flood depth is well below the building’s FFH. This raises some concerns as to how this may be true for all buildings and if using these DDFs for all buildings overestimates the flood risk. This study addresses this question by examining the effect of different DIPs of the appropriate DDF on flood risk. The AAL is evaluated for different DIP, specifically focusing on one-story, no-basement buildings using the USACE^21^ DDF. This evaluation is carried out within the context of different freeboard elevation.

The results of this study show the relationship between flood hazard and risk. One noteworthy finding is that BFD values had no impact on AAL calculation, given that buildings in SFHA typically conform to FEMA’s minimum elevation requirements, effectively fixing the FFH relative to BFD. This result emphasizes the critical role of the BFD in the context of flood risk assessment. The BFD serves as the national benchmark adopted not only by the National Flood Insurance Program (NFIP) but also by all federal agencies^31^. Within the home construction standards, areas characterized by wave heights of <1.5 feet—referred to as V Zones and Coastal A Zones in the United Sytates—traditionally mandated the placement of the FFH point at or above the BFD level^32^. This standard underwent modification in subsequent years^33^ to incorporate an additional 1.0 foot of elevation above BDF. Building at the BFD level essentially results in the AAL being consistent, irrespective of variations in BFD. Whether the BFD is 2 feet or 12 feet, as long as the FFH conforms to the BFD requirements, thus confirming that the non-exceedance probability of the FFH is fixed, the AAL calculation remains unchanged.

On the other hand, the results demonstrate the impact of *α* parameter on the estimation of AAL (%). This influence, however, shows variability depending on the value of DIP. These findings have led to the development of novel mathematical expressions that enhance the AAL prediction by accounting for different DIP scenarios. This innovation contributes to the adaptability of flood risk assessment in addressing a wide spectrum of potential flood scenarios. The integration of these newly developed equations in a comprehensive flood risk analysis tool represents a major advancement in flood risk management. This tool will allow practitioners and researchers to input critical parameters such as the *α* parameter and specific building characteristics. By doing so, it streamlines the AAL estimation process, simplifying what was once a complex and intensive task.

The impact of DIP on flood risk assessment is a pivotal aspect of this study. DIP plays a central role in shaping the flood risk patterns for buildings located within SFHAs. Figure 3 provides a visual representation of the relationship between DIP and flood risk patterns, underscoring the critical importance of considering this factor when evaluating flood risk and devising effective mitigation strategies. While prior research has implied the considerable impact of negative DIP on quantified risk^28^, this study goes a step further by providing detailed insights and visualizations. It effectively demonstrates that negative DIPs contribute to a significant amount of AAL (%) compared to positive DIPs. Thus, a major portion of AAL is already being accounted for in risk studies even before flood waters rise to the FFH. This finding highlights the importance of considering the DIP in flood risk assessment and warrants further attention in future studies.

**Fig. 5:**
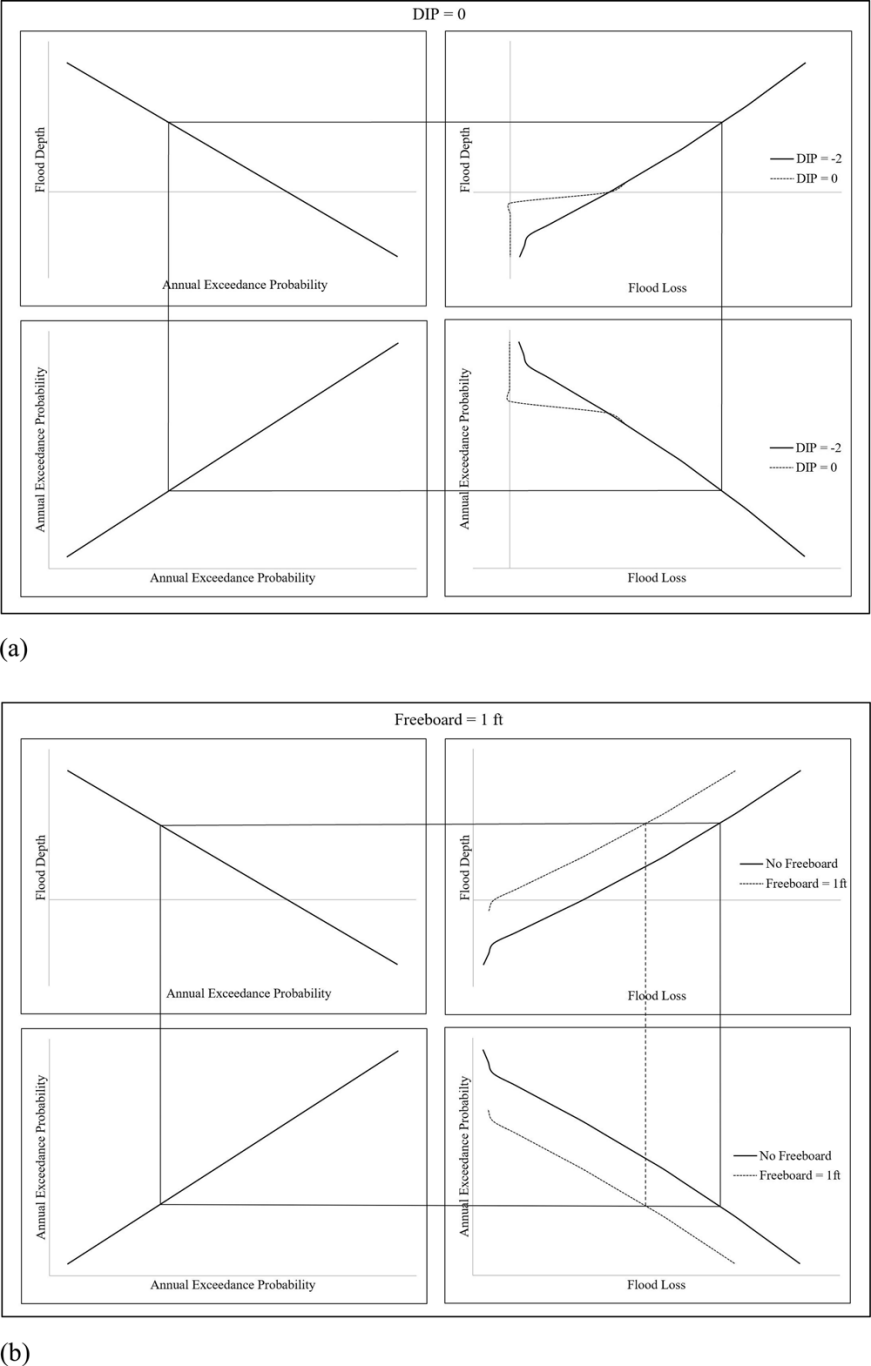
Interrelation between flood depth/probability and flood depth/loss to yield probability/loss relationship. **a** The shift of the curve due to changes in DIP from –2 to 0. **b** The shift of the curve due to a freeboard of +1 feet.

Moreover, regardless of the specific DIP values, results in this research strongly confirm that a considerable decrease in annual flood risk is achieved by freeboard increment. The results highlight the crucial role of freeboard as a flood mitigation measure for buildings. Even a small increase in freeboard, such as one foot, leads to a substantial reduction in flood risk. The AAL (%) curves presented in the Supplementary Fig. S.2 suggest that even when the flood damage occurs below the FFH, freeboard changes the curve direction to resemble one in which the flood damage occurs at or above the FFH (increasing with *α* parameter). Although freeboard shifts the AAL curve to be more similar to one with a higher DIP, the shift is not alike. Freeboard shifts the overall loss exceedance probability curve closer to the origin, which yields a lower area under the curve (i.e., AAL) where decreasing DIP changes the direction of the loss exceedance curve for higher exceedance probabilities (i.e., lower flood losses). Figure 5 shows the loss exceedance probability curve that is generated from USACE^21^ DDF^34^. Additional loss exceedance probability curves for different DIPs and freeboards are provided in Supplementary Figs. S.3, S.4.

To obtain a comprehensive understanding of the financial implications associated with varying DIP and freeboard values, the AAL estimates in terms of monetary values. While the range of DIP from –2 to 0 feet contributes to a relative reduction of 92% in mean AAL (%), it’s essential to recognize that even though the AAL (%) values appear small, the AAL ($) values exhibit distinct variations among different DIPs with each increment in freeboard. These variations underscore their substantial influence on financial assessments and considerations.

Although this research represents progress, further investigations in this area are needed. The current state of flood risk estimation still has inherent limitations that lead to either underestimating or overestimating actual risk. Therefore, exploring the potential utilization of component-based functions, as suggested by Matthews et al.^35^, to generate building-specific DDFs could prove valuable in refining flood risk assessments. Moreover, it’s crucial to acknowledge that the study presented here has some limitations that may be addressed in future work. This study is limited to USACE^21^ DDF for one-story, no-basement buildings located inside the SFHA. This work can be expanded to multi-story buildings with basements that are located in the V-zone, or outside the SFHA (i.e., shaded and unshaded X zone). Different sources of DDFs should also be explored to check the AAL variations to better specify the appropriate DIP for each DDF for risk studies. Despite these limitations, the study presented here is comprehensive in capturing the spatial heterogeneity of buildings. If the USACE^21^ DDF is applicable to a building with a known hazard, the analysis here can be used to generate flood risk insights for that building. Future research can further demonstrate the impacts of DIPs on AAL by using real-world case studies.

## Methods

The AAL is estimated using a Monte Carlo simulation^29^ for different DIPs of one-story, no-basement building USACE^21^ DDF and freeboard scenarios. The flood hazard is quantified using a Gumbel extreme value distribution^27,30,36^ and Monte Carlo simulations were conducted to estimate AAL with relation to Gumbel parameters for buildings located in the SFHA.

### Flood hazard quantification

To estimate the annual flood hazard occurrence probability at the individual building level, the Gumbel extreme value distribution function is used, with special attention given to the location (*μ*) and scale (*α*) parameters. The Gumbel extreme value distribution stands as one of the most widely embraced probability functions for flood peak prediction^37,38^ and flood frequency analysis, particularly in the calculation of return periods^39^. This distribution has shown itself to be more fitting for these purposes than other distributions like the generalized extreme value, Log Pearson type III, and log-normal distributions^40^.

The cumulative distribution function (CDF) of this distribution is the annual probability that a stochastic variable *X* is less than or equal to a flood event of depth *D* (annual non-exceedance probability), and is written as:

1 $$F\left(D\right)=P(X\le D)=\exp \left[-\exp \left(-\left(\frac{D-\mu }{\alpha }\right)\right)\right]$$

Solving the CDF yields the quantile of the distribution:

2 $${D=F}^{-1}\left(F\left(D\right)\right)=\mu -\alpha (\mathrm{ln}(-\mathrm{ln}(p)))$$

where $p=P(X\le D)$. The annual exceedance probability (AEP) of the flood event with depth *D* is ($1-p$).

### Depth-damage function

**Fig. 6:**
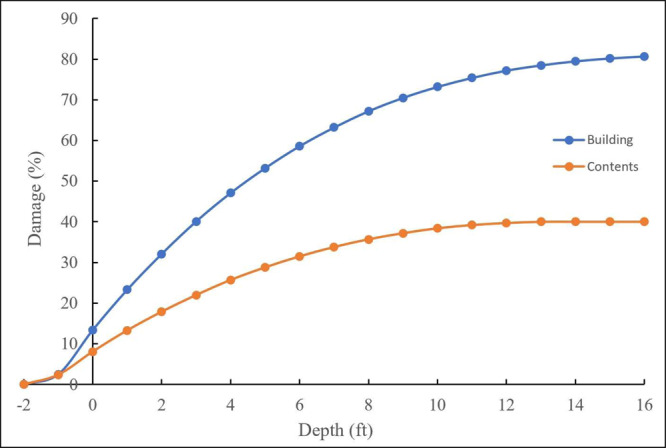
USACE^21^ DDF for a one-story home without basement.

Selecting the appropriate DDF is a pivotal decision in flood loss assessment, and by extension, in risk evaluation. The effective DDF should effectively show the relationship between ${\hat{D}}_{{S}_{i}}$ and the corresponding extent of damage^28^. For the purposes of this study, the USACE^21^ DDFs are employed to delineate the relationship between building (or contents) damage and ${\hat{D}}_{{S}_{i}}$. This choice was made primarily for the purpose of methodological demonstration, as these DDFs rely on generic data spanning the years 1996 to 2001. It’s noteworthy that DDFs such as USACE^22^ incorporate additional factors like flood duration and foundation type, making them suitable substitutes in scenarios where more detailed input data are available. USACE DDFs typically consider damage below the FFH by attributing losses at negative flood depths for buildings that lack a basement, as illustrated in Fig. 6. For USACE^21^ DDF, the DIP is at –2 feet below the FFH of a building. The damage is referred to as the percent of BRV. This study considers three DIP cases: below FFH (i.e., –2, –1, –0.5), at FFH (i.e., 0), and above FFH (i.e., 0.5, 1).

### AAL estimation

AAL is the area under the loss exceedance probability curve, and is calculated by integrating the loss function across the range of flood probabilities (Eq. 4),

3 $${AAL}=\mathop{\int}\nolimits_{{{\rm{P}}}_{\min }}^{{{\rm{P}}}_{\max }}L\left(P\right){dP}$$

where ${{\rm{P}}}_{\min }$ corresponds to the lowest exceedance probability and the ${{\rm{P}}}_{\max }$ corresponds to the highest exceedance probability. The methodology to estimate the loss function $L\left(P\right)$ in terms of a DIP of DDF is presented in Gnan et al.^27^. To estimate building-specific AAL, Monte Carlo simulation of *N* flood events is conducted^29^. In this study, 50,000 flood events for each Monte Carlo simulation are generated. The simulation process generates a random annual non-exceedance probability ($\hat{{p}_{i}}$) value between 0 and 1 for each run i, such that

4 $$\hat{{p}_{i}}={random}(0,1)$$

The USACE^21^ DDF is used to estimate flood loss by relating flood depth above the first-floor (${\hat{D}}_{{S}_{i}}$) to the damage as a percentage of BRV. The ${\hat{D}}_{{S}_{i}}$ is estimated using Eq. 5, where FFH is the first-floor height above the ground and FB is the freeboard height and ${\hat{D}}_{i}$ is the flood depth for each simulated flood event.

5 $${\hat{D}}_{{S}_{i}}={\hat{D}}_{i}-({FFH}+{FB})$$

The ${\hat{D}}_{i}$ is estimated using Eq. 2 by utilizing the probability derived from Eq. 4, along with the Gumbel parameters. Following this, Eq. 5 is employed to calculate the ${\hat{D}}_{{S}_{i}}$ value. The relationship between ${D}_{s}$ and the percent of damage is described using the DDF. The ${\hat{D}}_{{S}_{i}}$ is then input to the loss function to estimate flood loss as a percentage of the building replacement value (${Loss}( \% )$) if ${\hat{D}}_{{S}_{i}}$ exceeds the selected DIP; otherwise, the loss is assumed to be zero. The flood loss values from these *N* runs are then averaged to estimate the AAL (%) (Eq. 6).

6 $${AAL}( \% )=\frac{1}{N}\mathop{\sum }\limits_{i=1}^{N}{{Loss}( \% )}_{i}$$

### Data generation

In the United States, the regulatory standard for home construction has typically been to situate the top of the first floor at the base flood elevation (BFE) in areas where wave heights are <1.5 feet. The standard was later modified^33^ to include an additional 1.0 feet of elevation above the BFE. Here, flood depth is used instead of flood elevation, and base flood elevation is replaced with BFD (100-year return period flood depth).

As the buildings are located in the SFHA, the BFD will exceed zero. Substituting p into Eq. 2 for the 100-year return period, for which BFD is assumed to exceed zero in the SFHA yields Eqs. 7 and 8.

7 $${BFD}=\mu -\alpha \left(\mathrm{ln}\left(-\mathrm{ln}\left(1-\frac{1}{100}\right)\right)\right)$$

8 $$\mu ={BFD}-4.6* \alpha$$

The *α* parameter, which represents the scale parameter, should be positive. The study conducted by Al Assi et al.^41^ determine the upper limit for *α* to be 4.60, based on the assumption that the upper limit of this parameter occurs in coastal areas. Here, a range of *α* parameter is selected where the starting value of *α* is 0.05, and it increases incrementally with an increment of 0.05 up to 1.5. The corresponding *μ* parameters are estimated using Eq. 8, where the BFD values were taken as 0.1, 1, 2, 3, 4, 6, 8, 10, 12, and 15 feet.

The ${D}_{s}$ start at the DIP of DDF. Monte Carlo simulations are performed for DIPs at –2, –1, –0.5, 0, 0.5, and 1 foot when the building is situated at the BFD (the “no freeboard” scenario) to evaluate the AAL as a percentage of BRV. The difference in AAL (%) that results from moving from one DIP to another is provided to show the effect of DIP selection in AAL evaluation. Additional simulations are conducted using the same DIP cases to estimate AAL for freeboard scenarios +1, +2, +3, and +4 feet above the BFD to study the impact of freeboard selection on flood risk reduction.

### Supplementary information

Supplementary Materials

## Data Availability

The Python script and raw data supporting the conclusions of this article are provided in the Supplementary document.

## References

[1] Pistrika A, Tsakiris G, Nalbantis I (2014). Flood depth-damage functions for built environment. Environ. Process..

[2] Kim SH, Kim BS, Lee CH, Chung JH (2014). Development of depth-damage function by investigating flooded area with focusing on building damage. J. Korea Water Resour. Assoc..

[3] Middelmann‐Fernandes MH (2010). Flood damage estimation beyond stage–damage functions: an Australian example. J. Flood Risk Manag..

[4] Notaro V (2014). The effect of damage functions on urban flood damage appraisal. Procedia Eng..

[5] Wing OEJ (2022). Inequitable patterns of US flood risk in the Anthropocene. Nat. Clim. Change.

[6] Huizinga, J., De Moel, H., & Szewczyk, W. Global flood depth-damage functions: Methodology and the database with guidelines. EUR 28552 EN, Publications Office of the European Union, Luxembourg, 2017, 10.2760/16510, JRC105688 (2017).

[7] Romali, N. S., Sulaiman, M., Khushren, S. A., Yusop, Z., & Ismail, Z. Flood damage assessment: a review of flood stage–damage function curve. *In*: Abu Bakar, S., Tahir, W., Wahid, M., Mohd Nasir, S., Hassan, R. (eds). ISFRAM 2014. Springer, Singapore. 10.1007/978-981-287-365-1_13 (2015).

[8] McGrath H, Abo El Ezz A, Nastev M (2019). Probabilistic depth–damage curves for assessment of flood-induced building losses. Nat. Hazards.

[9] Martínez-Gomariz E, Forero-Ortiz E, Guerrero-Hidalga M, Castán S, Gómez M (2020). Flood depth‒damage curves for spanish urban areas. Sustainability.

[10] Sulong S, Romali NS (2022). The role of socio-economic and property variables in the establishment of flood depth-damage curve for the data-scarce area in Malaysia. Urban Water J..

[11] Naumann, T., Nikolowski, J., & Golz, S. Synthetic depth-damage functions–a detailed tool for analysing flood resilience of building types. In Road map towards a flood resilient urban environment, Final conference of the COST action C (Vol. 22). (2009).

[12] Velasco M, Cabello À, Russo B (2016). Flood damage assessment in urban areas. Application to the Raval district of Barcelona using synthetic depth damage curves. Urban Water J..

[13] Neubert M, Naumann T, Hennersdorf J, Nikolowski J (2014). The geographic information system‐based flood damage simulation model HOWAD. J. Flood Risk Manag..

[14] Davis, S. A., & Skaggs, L. L. Catalog of residential depth-damage functions used by the army corps of engineers in flood damage estimation. USACE Water resource Support Center, Institute for Water Resources, VA. https://apps.dtic.mil/sti/pdfs/ADA255462.pdf (1992).

[15] Lee CH, Kim SH, Hwang SB, Kim GH (2017). A study on development of flood depth-damage functions focused on school buildings. J. Korea Water Resour. Assoc..

[16] FEMA. Coastal construction manual. Washington, DC: Federal Emergency Management Agency. (2005)

[17] Wing OE, Pinter N, Bates PD, Kousky C (2020). New insights into US flood vulnerability revealed from flood insurance big data. Nat. Commun.

[18] FEMA. Multi-hazard loss estimation methodology, flood model, HAZUS, technical manual, developed by the Department of Homeland Security, Emergency Preparedness and Response Directorate, FEMA, Mitigation Division, Washington, D.C., under a contract with the National Institute of Building Sciences, Washington, D.C. Available at: https://www.fema.gov/sites/default/files/2020-09/fema_hazus_flood-model_technical-manual_2.1.pdf (2003).

[19] FEMA. (2004). Analysis of depth vs. damage relationships used in FEMA’s benefit-cost analysis.

[20] USACE. Economic Guidance Memorandum (EGM) 01-03, Generic Depth Damage Relationships. Washington, DC: US Army Corps of Engineers. Retrieved from https://planning.erdc.dren.mil/toolbox/library/EGMs/egm01-03.pdf (2000)

[21] USACE. Economic guidance memorandum 04-01: generic depth-damage relationships for residential structures with basements. US Army Corps of Engineers. Available at: https://planning.erdc.dren.mil/toolbox/library/EGMs/egm04-01.pdf (2003).

[22] USACE. Depth-damage relationships for structures, contents, and vehicles and content-to-structure value ratios (CSVR) in support of the Donaldsville to the Gulf, Louisiana, feasibility study. Washington, DC: US Army Corps of Engineers. https://www.mvn.usace.army.mil/Portals/56/docs/PD/Donaldsv-Gulf.pdf (2006).

[23] USACE. North Atlantic coast comprehensive study: resilient adaptation to increasing risk. Washington, DC: US Army Corps of Engineers. https://www.nad.usace.army.mil/Portals/40/docs/NACCS/10A_PhysicalDepthDmgFxSummary_26Jan2015.pdf (2015).

[24] FIA. Depth-percent damage curves. Federal Insurance Administration, US Department of Housing and Urban Development. (1974).

[25] Nofal OM, van de Lindt JW, Do TQ (2020). Multi-variate and single-variable flood fragility and loss approaches for buildings. Reliab. Eng. Syst. Saf..

[26] FEMA (2022). Discount Explanation Guide. https://www.fema.gov/sites/default/files/documents/fema_discount-Explanation-Guide.pdf (2022).

[27] Gnan E (2022). Improved building-specific flood risk assessment and implications for depth-damage function selection. Front. Water..

[28] Al Assi A, Mostafiz RB, Friedland CJ, Rahim MA, Rohli RV (2023). Flood risk assessment for residences at the neighborhood scale by owner/occupant type and first-floor height. Front. Big Data.

[29] Gnan E (2022). Economically optimizing elevation of new, single-family residences for flood mitigation via life-cycle benefit-cost analysis. Front. Environ. Sci..

[30] Mostafiz RB (2022). A data-driven spatial approach to characterize the flood hazard. Front. Big Data.

[31] FEMA. Floodplain management requirements a study guide and desk reference for local officials. Available at: https://mdfloodmaps.net/pdfs/Reduce_Your_Risk/FEMA_480_StudyGuide_DeskReference.pdf (2005).

[32] ASCE. (2005). Flood resistant design and construction.

[33] ASCE. (2014). Flood resistant design and construction.

[34] Lekuthai A, Vongvisessomjai S (2001). Intangible flood damage quantification. Sustain. Water Resour. Manag..

[35] Matthews E, Friedland C, Alsadi A (2021). Customising flood damage functions to estimate the carbon footprint of flood-related home repairs. J. Flood Risk Manag..

[36] Al Assi A (2023). Cost-effectiveness of federal CDBG-DR road home program mitigation assistance in Jefferson Parish, Louisiana. Nat. Hazards.

[37] Patel, M. B. Flood frequency analysis using Gumbel distribution method at Garudeshwar Weir, Narmada Basin. *Int. J. Trend Res. Dev.* 7(1). Available at: http://www.ijtrd.com/papers/IJTRD21899.pdf (2020).

[38] Parhi PK (2018). Flood management in Mahanadi Basin using HEC-RAS and Gumbel’s extreme value distribution. J. Inst. Eng.: A.

[39] Singh P, Sinha VSP, Vijhani A, Pahuja N (2018). Vulnerability assessment of urban road network from urban flood. Int. J. Disaster Risk Reduct..

[40] Onen F, Bagatur T (2017). Prediction of flood frequency factor for Gumbel distribution using regression and GEP model. Arab. J. Sci. Eng..

[41] Al Assi A, Mostafiz RB, Friedland CJ, Rohli RV, Rahim MA (2023). Homeowner flood risk and risk reduction from home elevation between the limits of the 100-and 500-year floodplains. Front. Earth Sci..

