# Supplementary material for: Effects of damage initiation points of depth-damage function on flood risk assessment
Source: NPJ Nat Hazards. 2024 May 2;1(1):6. doi: 10.1038/s44304-024-00004-z (PMC11078111; doi:10.1038/s44304-024-00004-z)
Supplement: Supplementary file 1 — Supplementary Materials [file 44304_2024_4_MOESM1_ESM.docx]

Supplementary Materials

**Supplementary Table S.1.** The AAL (%) values in the “no freeboard” scenario

| $\alpha$ | AAL (%) (DIP –2) | AAL (%) (DIP –1) | AAL (%) (DIP –0.5) | AAL (%) (DIP 0) | AAL (%) (DIP 0.5) | AAL (%) (DIP 1) |
| --- | --- | --- | --- | --- | --- | --- |
| 0.05 | 11.2092 | 11.2092 | 11.2092 | 0.1481 | 0 | 0 |
| 0.1 | 9.0183 | 9.0183 | 7.3219 | 0.1533 | 0.0012 | 0 |
| 0.15 | 6.8275 | 6.8263 | 2.4044 | 0.1585 | 0.0079 | 0.0005 |
| 0.2 | 4.8095 | 4.2922 | 1.2016 | 0.1637 | 0.0163 | 0.0015 |
| 0.25 | 3.5125 | 2.3863 | 0.7903 | 0.1689 | 0.0298 | 0.0042 |
| 0.3 | 2.6629 | 1.4973 | 0.6088 | 0.174 | 0.0428 | 0.0103 |
| 0.35 | 2.0007 | 1.0655 | 0.5112 | 0.1791 | 0.0554 | 0.0158 |
| 0.4 | 1.4956 | 0.8291 | 0.4481 | 0.1842 | 0.0673 | 0.0216 |
| 0.45 | 1.1521 | 0.6886 | 0.4093 | 0.1892 | 0.0783 | 0.0308 |
| 0.5 | 0.928 | 0.5963 | 0.3821 | 0.1942 | 0.0903 | 0.0395 |
| 0.55 | 0.7794 | 0.5353 | 0.3606 | 0.1991 | 0.0994 | 0.049 |
| 0.6 | 0.6783 | 0.4921 | 0.3463 | 0.204 | 0.1104 | 0.0572 |
| 0.65 | 0.6062 | 0.4613 | 0.3345 | 0.2088 | 0.1187 | 0.0665 |
| 0.7 | 0.5536 | 0.4362 | 0.3281 | 0.2135 | 0.1265 | 0.0746 |
| 0.75 | 0.5144 | 0.4172 | 0.322 | 0.2182 | 0.1371 | 0.0829 |
| 0.8 | 0.4847 | 0.4026 | 0.3193 | 0.2229 | 0.1447 | 0.0912 |
| 0.85 | 0.4616 | 0.3919 | 0.3163 | 0.2275 | 0.1527 | 0.0997 |
| 0.9 | 0.4434 | 0.3828 | 0.3133 | 0.2321 | 0.1592 | 0.1068 |
| 0.95 | 0.4291 | 0.376 | 0.3129 | 0.2366 | 0.168 | 0.1144 |
| 1 | 0.4178 | 0.3704 | 0.3124 | 0.2411 | 0.1742 | 0.1236 |
| 1.05 | 0.4087 | 0.3662 | 0.3131 | 0.2455 | 0.1799 | 0.1304 |
| 1.1 | 0.4014 | 0.3622 | 0.3144 | 0.2499 | 0.1857 | 0.1368 |
| 1.15 | 0.3956 | 0.3597 | 0.3155 | 0.2542 | 0.1915 | 0.1427 |
| 1.2 | 0.391 | 0.358 | 0.3166 | 0.2585 | 0.1965 | 0.1524 |
| 1.25 | 0.3874 | 0.3567 | 0.3194 | 0.2628 | 0.2029 | 0.1585 |
| 1.3 | 0.3845 | 0.3559 | 0.3203 | 0.267 | 0.2086 | 0.1645 |
| 1.35 | 0.3823 | 0.356 | 0.3215 | 0.2711 | 0.2153 | 0.1705 |
| 1.4 | 0.3807 | 0.3562 | 0.3237 | 0.2753 | 0.2213 | 0.1761 |
| 1.45 | 0.3796 | 0.3565 | 0.3254 | 0.2793 | 0.2266 | 0.184 |
| 1.5 | 0.379 | 0.3573 | 0.3279 | 0.2834 | 0.2329 | 0.191 |

**Supplementary Table S.2.** The AAL (%) values for +1 feet freeboard scenario

| $\alpha$ | AAL (%) (DIP –2) | AAL (%) (DIP –1) | AAL (%) (DIP –0.5) | AAL (%) (DIP 0) | AAL (%) (DIP 0.5) | AAL (%) (DIP 1) |
| --- | --- | --- | --- | --- | --- | --- |
| 0.05 | 2.0021 | 0.0324 | 0 | 0 | 0 | 0 |
| 0.1 | 1.5041 | 0.0381 | 0.0006 | 0 | 0 | 0 |
| 0.15 | 1.0062 | 0.0439 | 0.0038 | 0.0003 | 0 | 0 |
| 0.2 | 0.5597 | 0.0496 | 0.0082 | 0.001 | 0 | 0 |
| 0.25 | 0.3278 | 0.0553 | 0.0152 | 0.0026 | 0.0008 | 0 |
| 0.3 | 0.2289 | 0.061 | 0.0224 | 0.0064 | 0.0013 | 0.0005 |
| 0.35 | 0.1826 | 0.0666 | 0.0297 | 0.0101 | 0.0034 | 0.0011 |
| 0.4 | 0.1589 | 0.0723 | 0.0369 | 0.014 | 0.0049 | 0.0017 |
| 0.45 | 0.1462 | 0.0778 | 0.0439 | 0.0201 | 0.0089 | 0.0037 |
| 0.5 | 0.1395 | 0.0833 | 0.0515 | 0.026 | 0.0116 | 0.0046 |
| 0.55 | 0.1364 | 0.0888 | 0.0579 | 0.0325 | 0.0149 | 0.0073 |
| 0.6 | 0.1354 | 0.0941 | 0.0652 | 0.0384 | 0.0194 | 0.0112 |
| 0.65 | 0.1357 | 0.0995 | 0.0713 | 0.0451 | 0.0237 | 0.0138 |
| 0.7 | 0.137 | 0.1048 | 0.0774 | 0.0511 | 0.0301 | 0.0175 |
| 0.75 | 0.139 | 0.11 | 0.0846 | 0.0573 | 0.0359 | 0.0204 |
| 0.8 | 0.1415 | 0.1152 | 0.0906 | 0.0636 | 0.0427 | 0.0243 |
| 0.85 | 0.1445 | 0.1204 | 0.0968 | 0.07 | 0.0478 | 0.0283 |
| 0.9 | 0.1476 | 0.1255 | 0.1023 | 0.0757 | 0.053 | 0.0348 |
| 0.95 | 0.151 | 0.1305 | 0.1088 | 0.0817 | 0.0591 | 0.0387 |
| 1 | 0.1546 | 0.1355 | 0.1142 | 0.0887 | 0.0645 | 0.045 |
| 1.05 | 0.1583 | 0.1404 | 0.1195 | 0.0943 | 0.0704 | 0.051 |
| 1.1 | 0.1621 | 0.1453 | 0.1247 | 0.0997 | 0.076 | 0.0562 |
| 1.15 | 0.1659 | 0.1502 | 0.1298 | 0.1048 | 0.0817 | 0.0623 |
| 1.2 | 0.1698 | 0.155 | 0.1346 | 0.1122 | 0.0874 | 0.0662 |
| 1.25 | 0.1738 | 0.1597 | 0.1401 | 0.1174 | 0.0939 | 0.0726 |
| 1.3 | 0.1778 | 0.1644 | 0.1452 | 0.1226 | 0.0983 | 0.0776 |
| 1.35 | 0.1819 | 0.1691 | 0.1508 | 0.1279 | 0.1038 | 0.0831 |
| 1.4 | 0.1859 | 0.1737 | 0.156 | 0.1328 | 0.1104 | 0.0877 |
| 1.45 | 0.1899 | 0.1782 | 0.1608 | 0.1392 | 0.1157 | 0.0934 |
| 1.5 | 0.194 | 0.1827 | 0.1662 | 0.145 | 0.1213 | 0.0981 |

**Supplementary Table S.3.** The AAL (%) values for +2 feet freeboard scenario

| $\alpha$ | AAL (%) (DIP –2) | AAL (%) (DIP –1) | AAL (%) (DIP –0.5) | AAL (%) (DIP 0) | AAL (%) (DIP 0.5) | AAL (%) (DIP 1) |
| --- | --- | --- | --- | --- | --- | --- |
| 0.05 | 0.0013 | 0 | 0 | 0 | 0 | 0 |
| 0.1 | 0.0026 | 0 | 0 | 0 | 0 | 0 |
| 0.15 | 0.004 | 0.0001 | 0 | 0 | 0 | 0 |
| 0.2 | 0.0054 | 0.0003 | 0 | 0 | 0 | 0 |
| 0.25 | 0.007 | 0.0009 | 0.0004 | 0 | 0 | 0 |
| 0.3 | 0.0088 | 0.0022 | 0.0007 | 0.0003 | 0 | 0 |
| 0.35 | 0.011 | 0.0037 | 0.0018 | 0.0007 | 0.0004 | 0 |
| 0.4 | 0.0133 | 0.0056 | 0.0027 | 0.0011 | 0.0005 | 0 |
| 0.45 | 0.0159 | 0.0082 | 0.0049 | 0.0024 | 0.0009 | 0.0005 |
| 0.5 | 0.0189 | 0.011 | 0.0067 | 0.0031 | 0.0015 | 0.0011 |
| 0.55 | 0.022 | 0.0143 | 0.0088 | 0.0049 | 0.0024 | 0.0012 |
| 0.6 | 0.0254 | 0.0176 | 0.0116 | 0.0075 | 0.0039 | 0.0018 |
| 0.65 | 0.029 | 0.0213 | 0.0144 | 0.0094 | 0.0043 | 0.0024 |
| 0.7 | 0.0326 | 0.025 | 0.0182 | 0.012 | 0.0073 | 0.0046 |
| 0.75 | 0.0364 | 0.0289 | 0.022 | 0.0142 | 0.0098 | 0.005 |
| 0.8 | 0.0404 | 0.0329 | 0.0263 | 0.0171 | 0.0118 | 0.0067 |
| 0.85 | 0.0444 | 0.0371 | 0.03 | 0.0201 | 0.0145 | 0.0091 |
| 0.9 | 0.0484 | 0.0411 | 0.0338 | 0.0245 | 0.0166 | 0.012 |
| 0.95 | 0.0525 | 0.0454 | 0.0381 | 0.0276 | 0.0195 | 0.0142 |
| 1 | 0.0567 | 0.0498 | 0.0421 | 0.0321 | 0.0221 | 0.0159 |
| 1.05 | 0.0609 | 0.0541 | 0.0464 | 0.0365 | 0.0256 | 0.0191 |
| 1.1 | 0.0651 | 0.0583 | 0.0507 | 0.0406 | 0.0285 | 0.0205 |
| 1.15 | 0.0694 | 0.0626 | 0.055 | 0.0452 | 0.0337 | 0.0243 |
| 1.2 | 0.0736 | 0.0673 | 0.0594 | 0.0486 | 0.0367 | 0.0268 |
| 1.25 | 0.0779 | 0.0716 | 0.0642 | 0.0534 | 0.0414 | 0.0298 |
| 1.3 | 0.0822 | 0.076 | 0.0681 | 0.0575 | 0.0458 | 0.0329 |
| 1.35 | 0.0865 | 0.0803 | 0.0725 | 0.0619 | 0.0507 | 0.0384 |
| 1.4 | 0.0908 | 0.0846 | 0.0774 | 0.0658 | 0.0545 | 0.0417 |
| 1.45 | 0.0951 | 0.0892 | 0.0817 | 0.0704 | 0.0585 | 0.045 |
| 1.5 | 0.0993 | 0.0937 | 0.0862 | 0.0744 | 0.062 | 0.0499 |

**Supplementary Table S.4.** The AAL (%) values for +3 feet freeboard scenario

| $\alpha$ | AAL (%) (DIP –2) | AAL (%) (DIP –1) | AAL (%) (DIP –0.5) | AAL (%) (DIP 0) | AAL (%) (DIP 0.5) | AAL (%) (DIP 1) |
| --- | --- | --- | --- | --- | --- | --- |
| 0.05 | 0 | 0 | 0 | 0 | 0 | 0 |
| 0.1 | 0 | 0 | 0 | 0 | 0 | 0 |
| 0.15 | 0 | 0 | 0 | 0 | 0 | 0 |
| 0.2 | 0 | 0 | 0 | 0 | 0 | 0 |
| 0.25 | 0.0001 | 0 | 0 | 0 | 0 | 0 |
| 0.3 | 0.0003 | 0.0001 | 0 | 0 | 0 | 0 |
| 0.35 | 0.0006 | 0.0003 | 0.0002 | 0 | 0 | 0 |
| 0.4 | 0.0011 | 0.0005 | 0.0003 | 0 | 0 | 0 |
| 0.45 | 0.0017 | 0.001 | 0.0005 | 0.0003 | 0 | 0 |
| 0.5 | 0.0025 | 0.0014 | 0.0009 | 0.0007 | 0.0004 | 0 |
| 0.55 | 0.0035 | 0.0022 | 0.0014 | 0.0008 | 0.0005 | 0.0005 |
| 0.6 | 0.0047 | 0.0033 | 0.0023 | 0.0012 | 0.0009 | 0.0005 |
| 0.65 | 0.0061 | 0.0045 | 0.0027 | 0.0017 | 0.0011 | 0.0006 |
| 0.7 | 0.0077 | 0.0059 | 0.0044 | 0.0031 | 0.0016 | 0.0012 |
| 0.75 | 0.0095 | 0.0073 | 0.0059 | 0.0035 | 0.0022 | 0.0018 |
| 0.8 | 0.0114 | 0.009 | 0.0073 | 0.0047 | 0.0035 | 0.0019 |
| 0.85 | 0.0134 | 0.0109 | 0.0091 | 0.0064 | 0.0043 | 0.0026 |
| 0.9 | 0.0156 | 0.0131 | 0.0107 | 0.0084 | 0.0047 | 0.0042 |
| 0.95 | 0.0179 | 0.0153 | 0.0127 | 0.01 | 0.0065 | 0.005 |
| 1 | 0.0204 | 0.0178 | 0.0147 | 0.0115 | 0.0086 | 0.0053 |
| 1.05 | 0.0231 | 0.0205 | 0.017 | 0.0138 | 0.0107 | 0.0061 |
| 1.1 | 0.0259 | 0.0232 | 0.0192 | 0.0152 | 0.0126 | 0.0084 |
| 1.15 | 0.0288 | 0.0262 | 0.0225 | 0.0179 | 0.0141 | 0.0104 |
| 1.2 | 0.0317 | 0.0289 | 0.0249 | 0.0199 | 0.0161 | 0.0128 |
| 1.25 | 0.0348 | 0.032 | 0.0281 | 0.0222 | 0.0182 | 0.0149 |
| 1.3 | 0.0379 | 0.0351 | 0.0313 | 0.0247 | 0.0196 | 0.0162 |
| 1.35 | 0.0411 | 0.0383 | 0.0348 | 0.0286 | 0.0225 | 0.0184 |
| 1.4 | 0.0443 | 0.0414 | 0.0378 | 0.0312 | 0.0247 | 0.0206 |
| 1.45 | 0.0475 | 0.0447 | 0.0409 | 0.034 | 0.0273 | 0.022 |
| 1.5 | 0.0509 | 0.048 | 0.0439 | 0.0376 | 0.0301 | 0.0243 |

**Supplementary Table S.5.** The AAL (%) values for +4 feet freeboard scenario

| $\alpha$ | AAL (%) (DIP –2) | AAL (%) (DIP –1) | AAL (%) (DIP –0.5) | AAL (%) (DIP 0) | AAL (%) (DIP 0.5) | AAL (%) (DIP 1) |
| --- | --- | --- | --- | --- | --- | --- |
| 0.05 | 0 | 0 | 0 | 0 | 0 | 0 |
| 0.1 | 0 | 0 | 0 | 0 | 0 | 0 |
| 0.15 | 0 | 0 | 0 | 0 | 0 | 0 |
| 0.2 | 0 | 0 | 0 | 0 | 0 | 0 |
| 0.25 | 0 | 0 | 0 | 0 | 0 | 0 |
| 0.3 | 0 | 0 | 0 | 0 | 0 | 0 |
| 0.35 | 0 | 0 | 0 | 0 | 0 | 0 |
| 0.4 | 0.0001 | 0 | 0 | 0 | 0 | 0 |
| 0.45 | 0.0002 | 0.0001 | 0 | 0 | 0 | 0 |
| 0.5 | 0.0004 | 0.0003 | 0.0002 | 0 | 0 | 0 |
| 0.55 | 0.0006 | 0.0004 | 0.0003 | 0.0003 | 0 | 0 |
| 0.6 | 0.0009 | 0.0006 | 0.0005 | 0.0003 | 0 | 0 |
| 0.65 | 0.0013 | 0.0009 | 0.0007 | 0.0004 | 0.0004 | 0 |
| 0.7 | 0.0019 | 0.0015 | 0.001 | 0.0008 | 0.0005 | 0.0005 |
| 0.75 | 0.0025 | 0.0019 | 0.0014 | 0.0012 | 0.0009 | 0.0006 |
| 0.8 | 0.0032 | 0.0025 | 0.0022 | 0.0014 | 0.0011 | 0.0006 |
| 0.85 | 0.004 | 0.0034 | 0.0028 | 0.0019 | 0.0016 | 0.0012 |
| 0.9 | 0.005 | 0.0044 | 0.0032 | 0.0029 | 0.0017 | 0.0013 |
| 0.95 | 0.0062 | 0.0055 | 0.0043 | 0.0036 | 0.0023 | 0.0019 |
| 1 | 0.0074 | 0.0065 | 0.0055 | 0.0039 | 0.0036 | 0.002 |
| 1.05 | 0.0088 | 0.0079 | 0.0069 | 0.0046 | 0.0043 | 0.0027 |
| 1.1 | 0.0103 | 0.0091 | 0.0083 | 0.0062 | 0.0047 | 0.0033 |
| 1.15 | 0.0119 | 0.0107 | 0.0095 | 0.0075 | 0.005 | 0.005 |
| 1.2 | 0.0135 | 0.0122 | 0.011 | 0.0093 | 0.0065 | 0.0054 |
| 1.25 | 0.0153 | 0.0138 | 0.0125 | 0.0109 | 0.0077 | 0.0057 |
| 1.3 | 0.0171 | 0.0155 | 0.0138 | 0.012 | 0.0094 | 0.006 |
| 1.35 | 0.0191 | 0.0176 | 0.0158 | 0.0137 | 0.0115 | 0.0082 |
| 1.4 | 0.0212 | 0.0196 | 0.0175 | 0.0155 | 0.0134 | 0.0101 |
| 1.45 | 0.0233 | 0.0216 | 0.0195 | 0.0168 | 0.0142 | 0.0116 |
| 1.5 | 0.0256 | 0.024 | 0.0215 | 0.0186 | 0.0157 | 0.0136 |


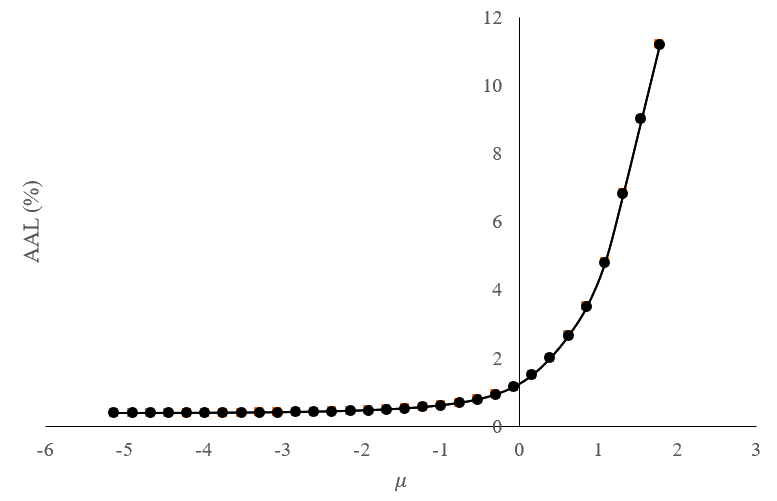

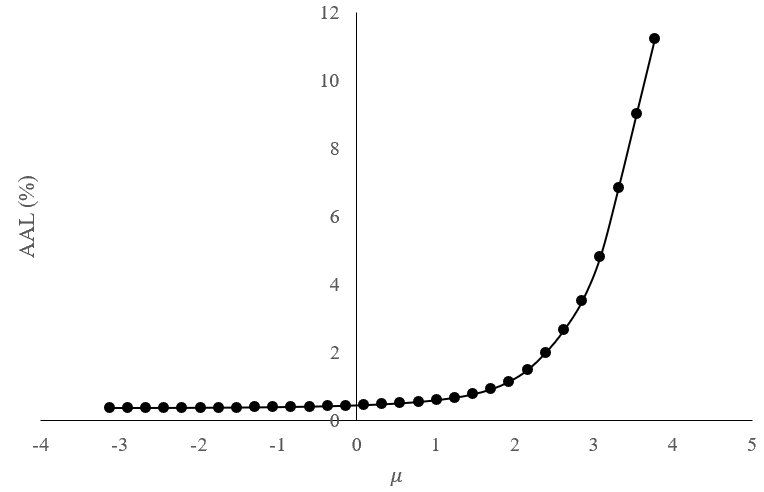


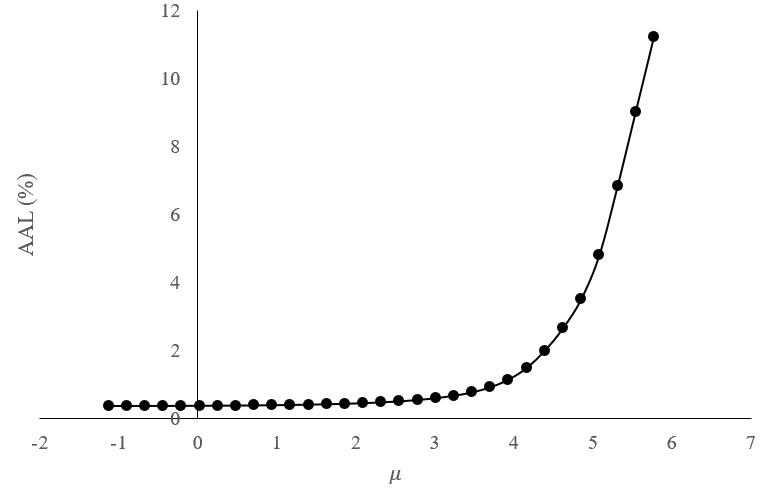

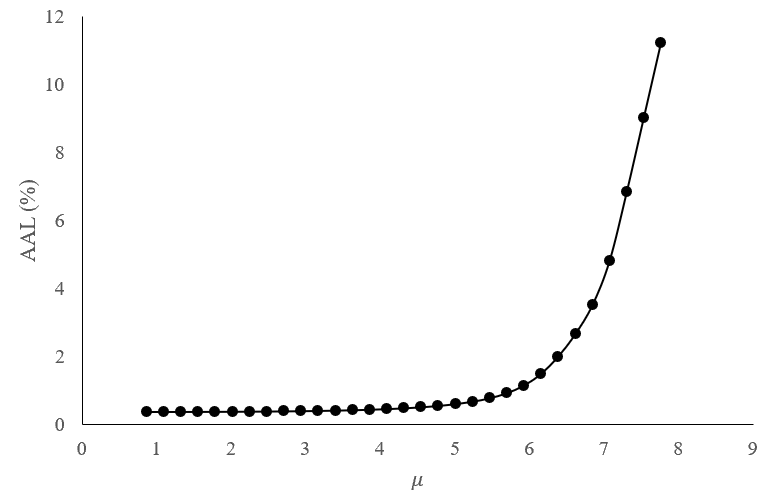


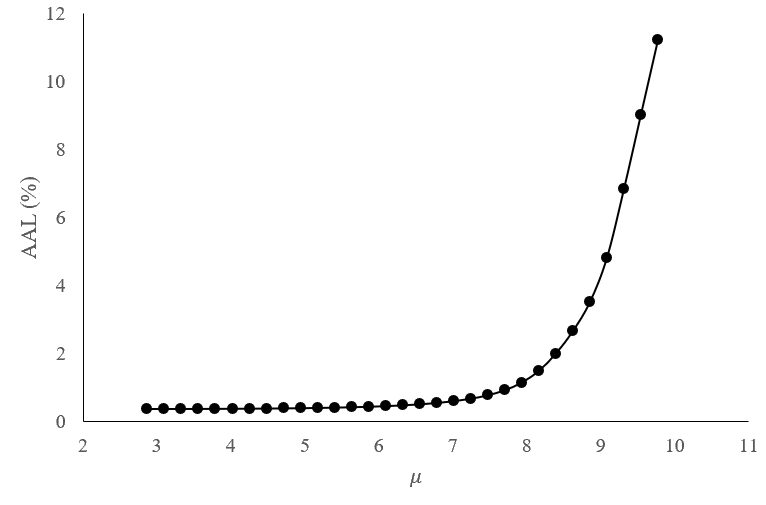


Supplementary Figure S.1. AAL (%) curves with respect to $\mu$ for BFDs of 2, 4, 6, 8, and 10 feet. The curve just shifts with BFD value. There is no change in AAL (%) values.


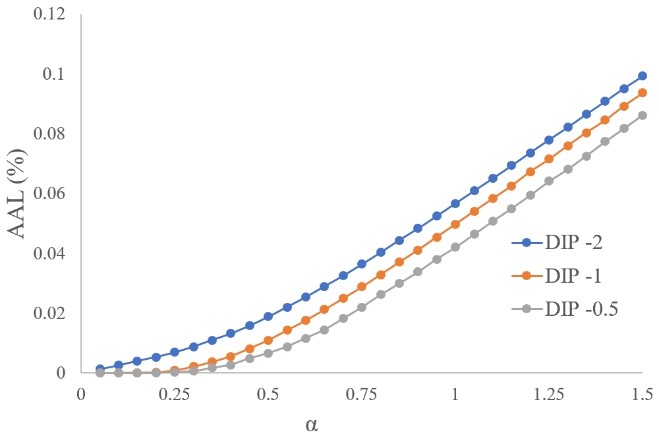

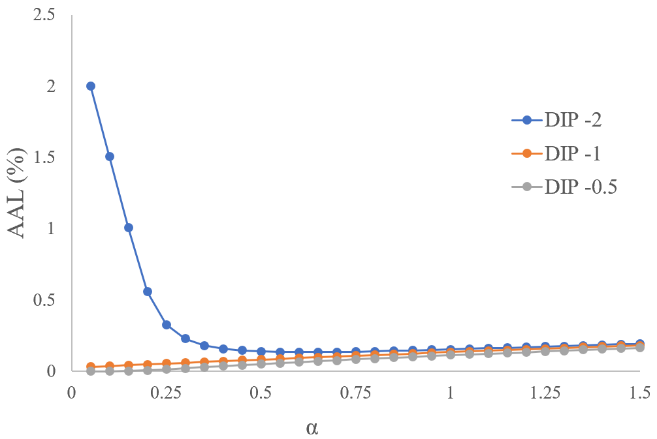
 (a) (b)


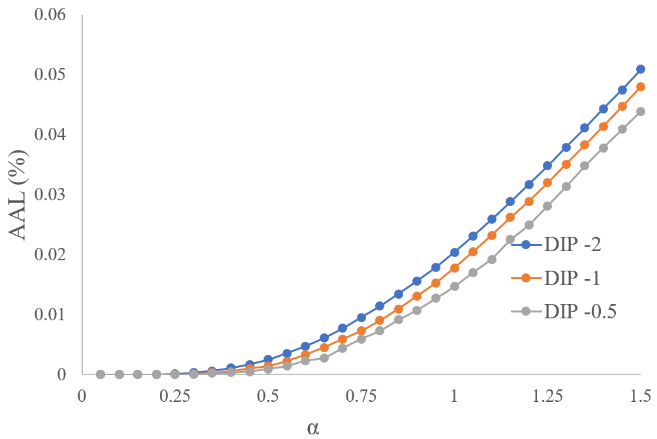

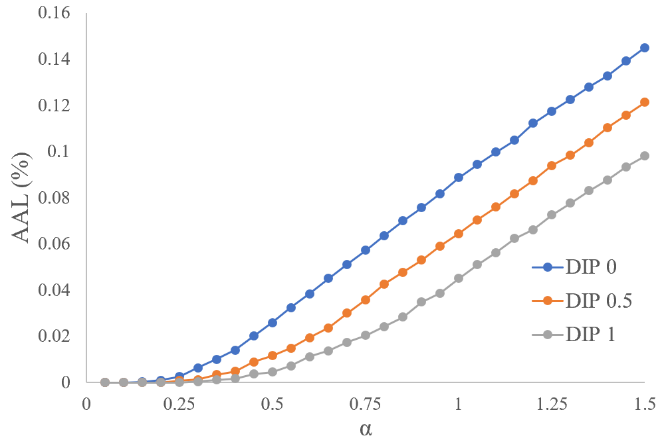


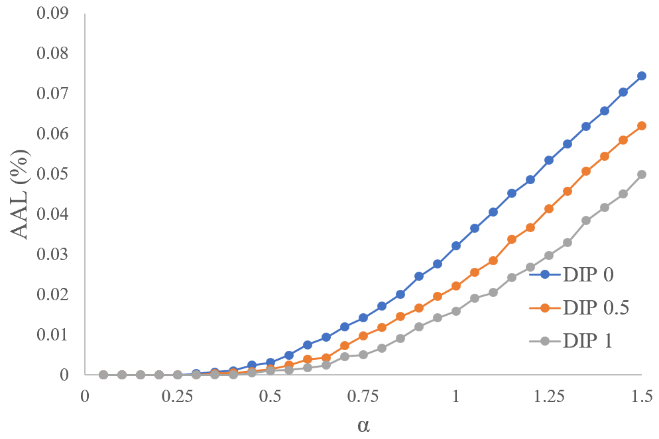

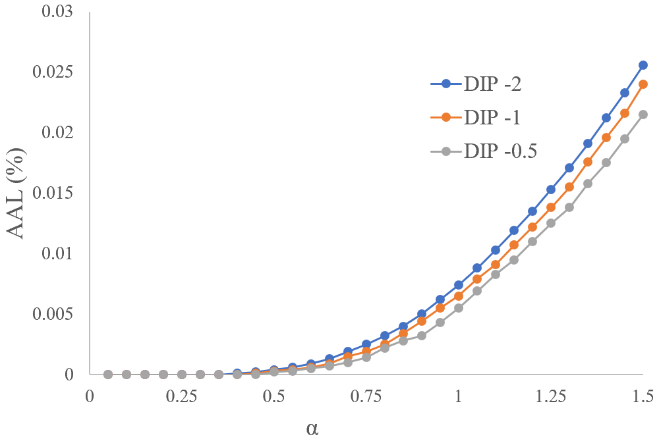
 (c) (d)

(e) (f)


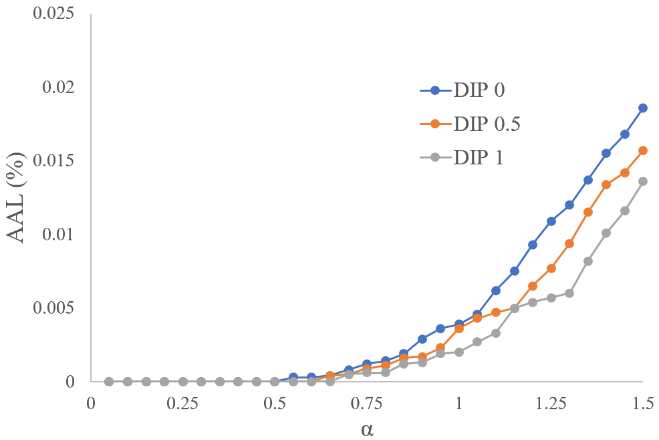

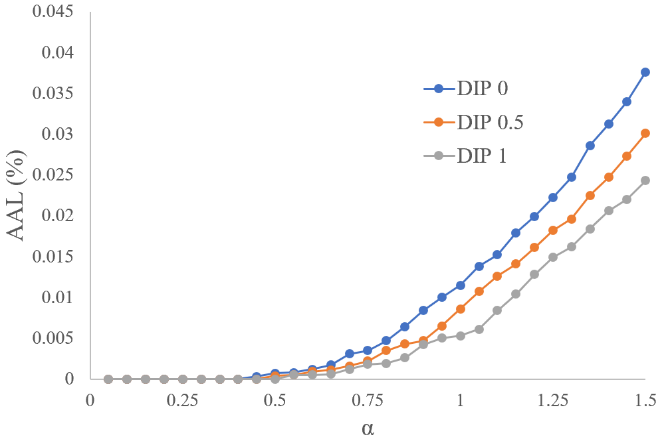
(g) (h)

Supplementary Figure S.2. AAL (%) curves with $\alpha$ parameter for different DIPs with increasing freeboard of +1 feet (a), +2 feet (b), +3 feet (c), and +4 feet (d).


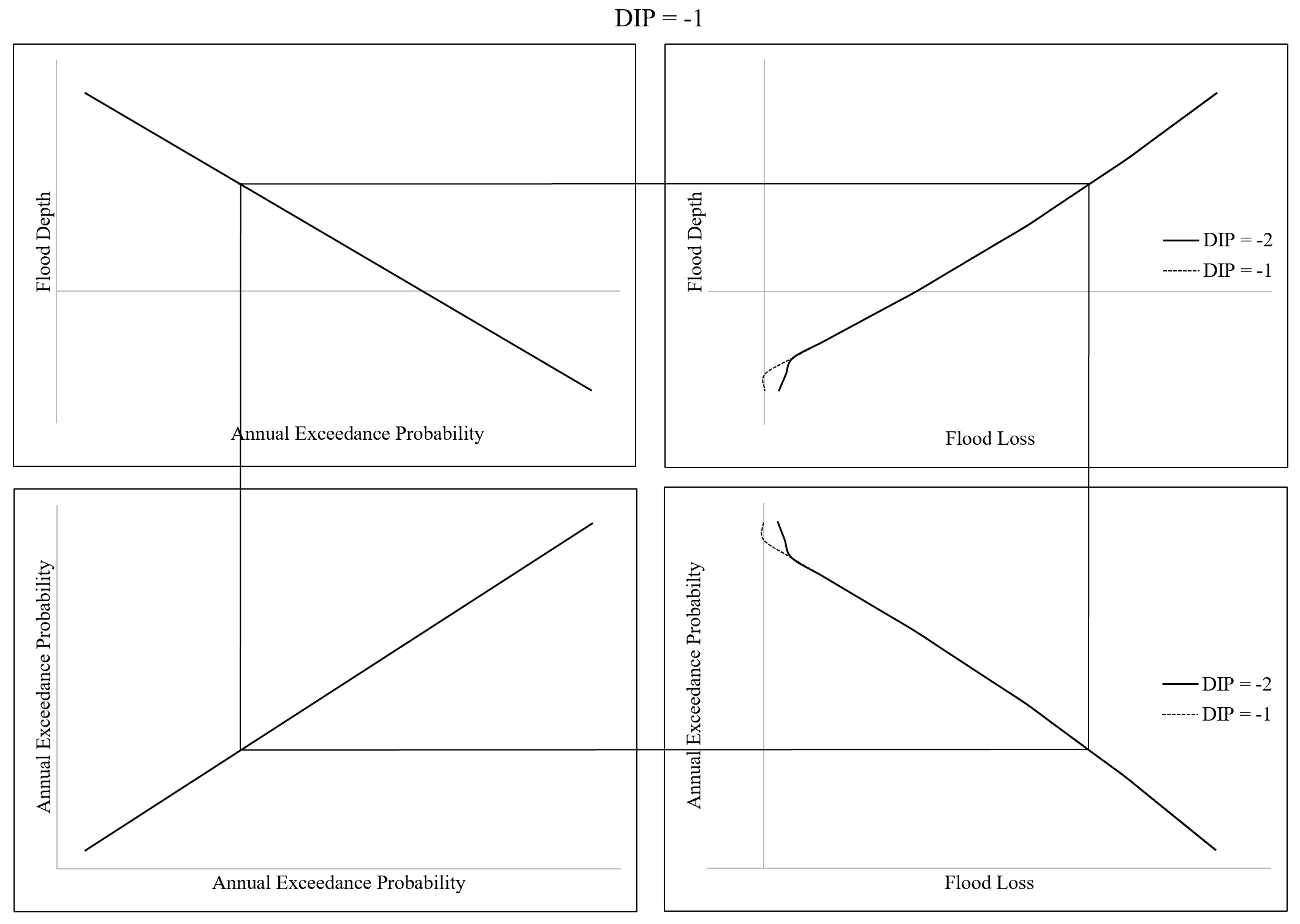


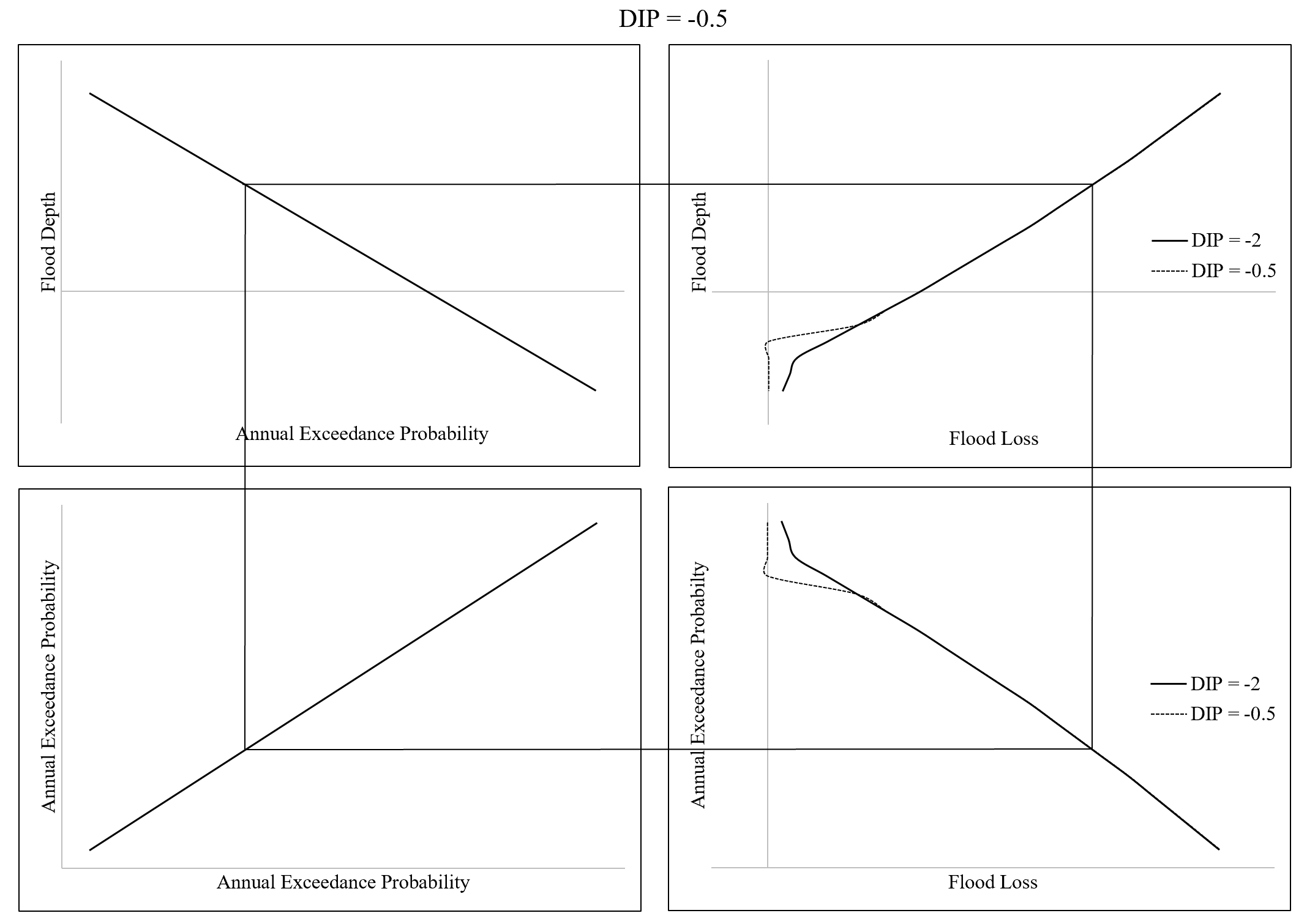


Supplementary Figure S.3. Interrelation between flood depth/probability and flood depth/loss to yield probability/loss relationship. The shift of the curve due to changes in DIPs.


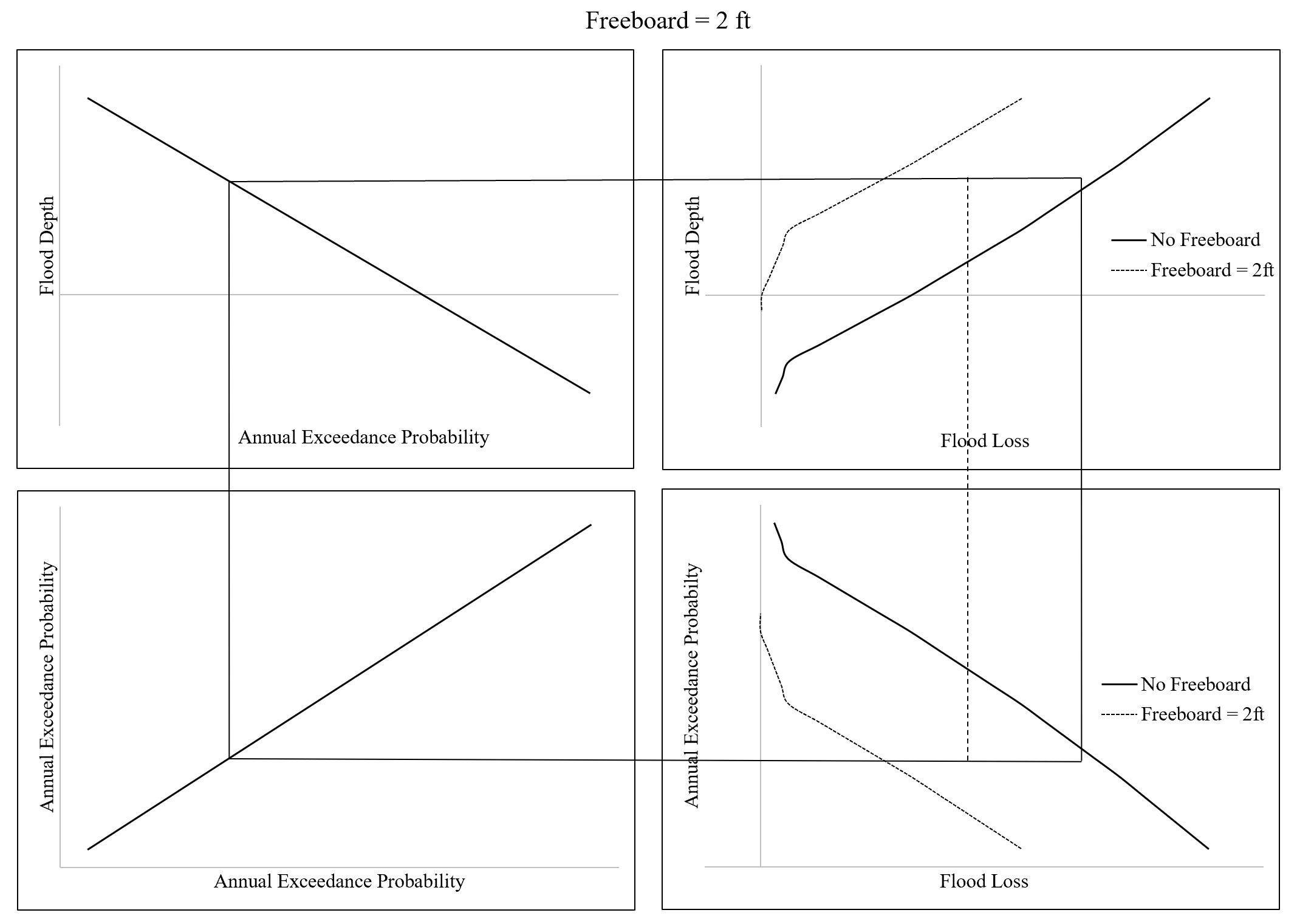


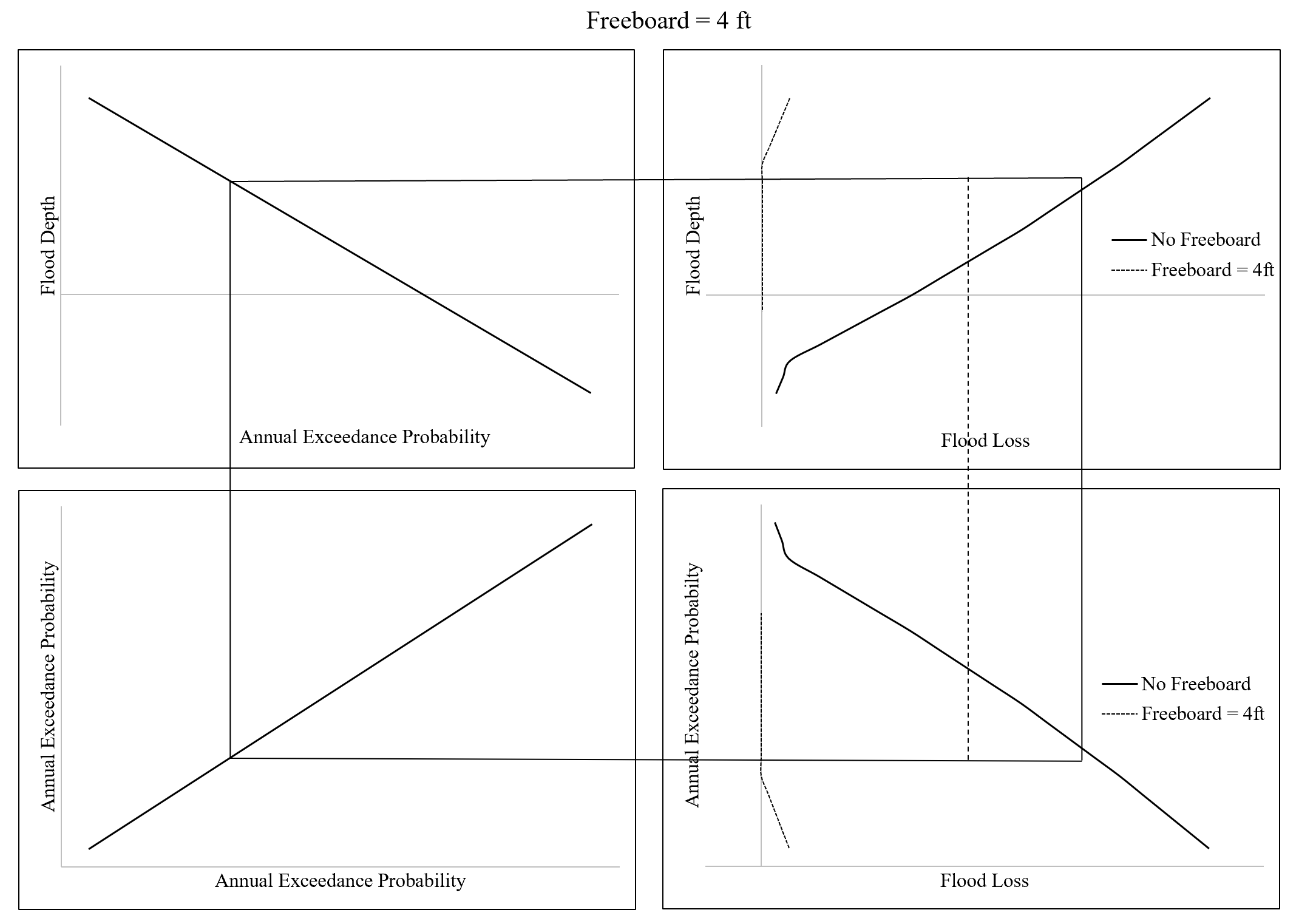


Supplementary Figure S.4. Interrelation between flood depth/probability and flood depth/loss to yield probability/loss relationship. The curve shifts with increasing freeboard.


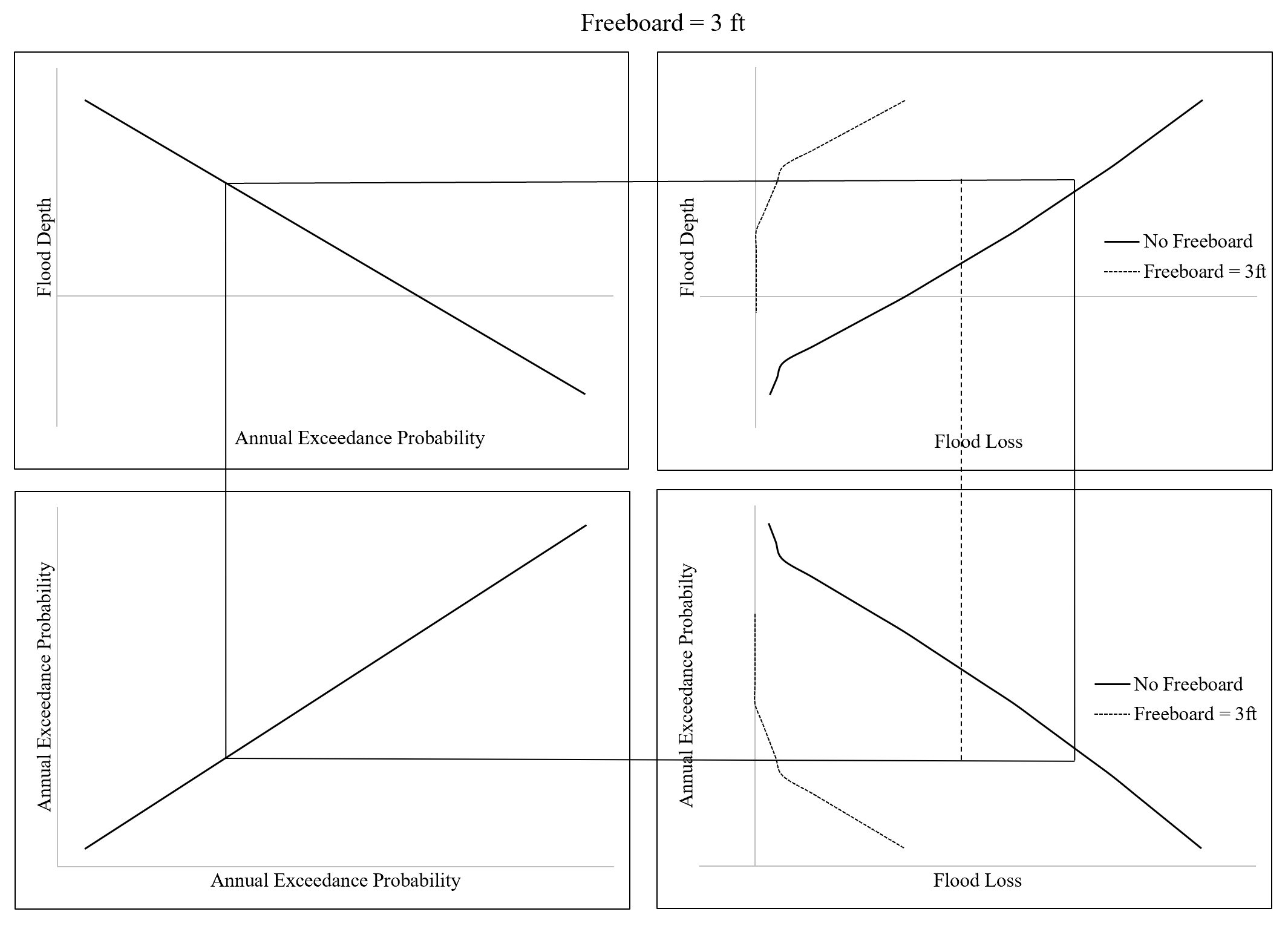


Supplementary Figure S.4. Interrelation between flood depth/probability and flood depth/loss to yield probability/loss relationship. The curve shifts with increasing freeboard.

**Python Script:**

#import libraries

import numpy as np

import random

import pandas as pd

#define the functions

def uo(bfe,a):

return bfe-4.6*a

def get_probability():

return (random.uniform(0,1))

def inv_flood_depth(x,u,a):

return u - a * np.log(-(np.log(1-x)))

def monte_carlo(intercept_u,slope_a,ffh,dip,ddf,num_samples=50000):

sum_of_str = 0

for i in range(num_samples):

x = get_probability()

flood_depth = inv_flood_depth(x,intercept_u,slope_a)

depth_above_ff = flood_depth - ffh

if depth_above_ff<dip:

str_dam = 0

else:

str_dam = np.interp(depth_above_ff,ddf[ddf.columns[0]],ddf[ddf.columns[1]])

sum_of_str += str_dam

return float(sum_of_str/num_samples)

#read the depth-damage function

ddf = pd.read_csv('USACE_2000.csv')

#provide the ddf with two columns, depth (ft) and damage (%)

#define parameters

bfds = [0,1,2,3,4,6,8,10,12,15]

dips = [-2,-1,-0.5, 0, 0.5, 1]

fbs= [0,1,2,3,4]

# run monte carlo simulation and save results

for fb in fbs:

risks = []

for bfd in bfds:

for dip in dips:

a = 0.05

u = round(u0(bfd,a),4)

random.seed(123)

aal = round(monte_carlo(u, a, bfd+fb, dip, ddf),4)

aals = []

u_s = []

a_s = []

aals.append(aal)

u_s.append(u)

a_s.append(a)

for i in range(0,30):

a = round(a + 0.05,4)

u = round(u0(bfd,a),4)

random.seed(123)

aal = round(monte_carlo(u, a, bfd+fb, dip, ddf),4)

aals.append(aal)

u_s.append(u)

a_s.append(a)

risks.append(aals)

df = pd.DataFrame(risks)

df1 = df.transpose()

df1.columns = dips

df1.to_csv("result_fb_"+str(fb)+"_bfd_"+str(bfd)+".csv",index=False)
